# Stromal lipid species dictate melanoma metastasis and tropism

**DOI:** 10.1016/j.ccell.2025.04.001

**Published:** 2025-04-24

**Authors:** Shilpa Gurung, Timothy Budden, Karthik Mallela, Benjamin Jenkins, Alex von Kriegsheim, Esperanza Manrique, David Millán-Esteban, Isabel Romero-Camarero, Fabio Amaral, Sarah Craig, Pedro Durao, Joanna Pozniak, Laura Stennett, Duncan Smith, Garry Ashton, Alex Baker, Kang Zeng, Gilbert Fruhwirth, Victoria Sanz-Moreno, Jair Marques, Albert Koulman, Jean-Christophe Marine, Tim C.P. Somervaille, Luisa Motta, Caroline Gaudy-Marqueste, Eduardo Nagore, Amaya Virós

**Affiliations:** 1 Skin Cancer and Ageing Lab, Cancer Research UK Manchester Institute, the University of Manchester, Manchester, UK; 2 Wellcome Trust - MRC Institute of Metabolic Science Metabolic Research Laboratories, Addenbrooke’s Hospital, Hills Road, Cambridge CB2 0QQ, UK; 3 Medical Research Council Human Nutrition Research, Elsie Widdowson Laboratory, University of Cambridge, Fulbourn Road, Cambridge CB1 9NL, UK; 4 CRUK Scotland Centre, Institute of Genetics and Cancer, The University of Edinburgh, Edinburgh EH4 2XU, UK; 5 School of Medicine, Universidad Católica de Valencia, San Vicente Mártir, 46001 Valencia, Spain; 6 Department of Dermatology, Fundación Instituto Valenciano de Oncología, 46009 Valencia, Spain; 7 Leukaemia Biology Laboratory, Cancer Research UK Manchester Institute, The University of Manchester, Manchester M20 4GJ, UK; 8 Laboratory for Molecular Cancer Biology, Center for Cancer Biology, VIB, Leuven, Belgium; 9 Department of Oncology, KU Leuven, Leuven, Belgium; 10 Imaging Therapies and Cancer Group, Comprehensive Cancer Centre, School of Cancer and Pharmaceutical Studies, King’s College London, Guy’s Campus, London, UK; 11 School of Biomedical Engineering and Imaging Sciences, King’s College London, St. Thomas’ Hospital, London, UK; 12 Proteomics, Cancer Research UK Manchester Institute, The University of Manchester, Manchester SK10 4TG, UK; 13 Histology, Cancer Research UK Manchester Institute, The University of Manchester, Manchester SK10 4TG, UK; 14 Visualisation, Irradiation & Analysis, Cancer Research UK Manchester Institute, The University of Manchester, Manchester SK10 4TG, UK; 15 Cytoskeleton and Metastasis Team, The Breast Cancer Now Toby Robins Research Centre Division of Breast Cancer Research, The Institute of Cancer Research, Chester Beatty Laboratories, London SW3 6JB, UK; 16 Centre for Tumour Microenvironment at Barts Cancer Institute, Queen Mary University of London, Charterhouse Square Campus, John Vane Science Centre, London, UK; 17 Department of Histopathology, Salford Royal NHS Foundation Trust, The University of Manchester, Manchester, UK; 18 Aix Marseille University, APHM, CRCM Inserm U1068, CNRS U7258, CHU Timone, Dermatology and Skin Cancer Department, Marseille, France; 19 NIHR Manchester Biomedical Research Centre, Manchester, UK; 20 Department of Dermatology, Salford Royal NHS Foundation Trust, The University of Manchester, Manchester, UK

## Abstract

Cancer cells adapt to signals in the tumor microenvironment (TME), but the TME cues that impact metastasis and tropism are still incompletely understood. We show that abundant stromal lipids from young subcutaneous adipocytes, including phosphatidylcholines, are taken up by melanoma cells, where they upregulate melanoma PI3K-AKT signaling, fatty acid oxidation, oxidative phosphorylation (OXPHOS) leading to oxidative stress, resulting in decreased metastatic burden. High OXPHOS melanoma cells predominantly seed the lung and brain; decreasing oxidative stress with antioxidants shifts tropism from the lung to the liver. By contrast, the aged TME provides fewer total lipids but is rich in ceramides, leading to lower OXPHOS and high metastatic burden. Aged TME ceramides taken up by melanoma cells activate the S1P-STAT3-IL-6 signaling axis and promote liver tropism. Inhibiting OXPHOS in the young TME or blocking the IL-6 receptor in the aged TME reduces the age-specific patterns of metastasis imposed by lipid availability.

## Graphical abstract

**Graphic abstract:**
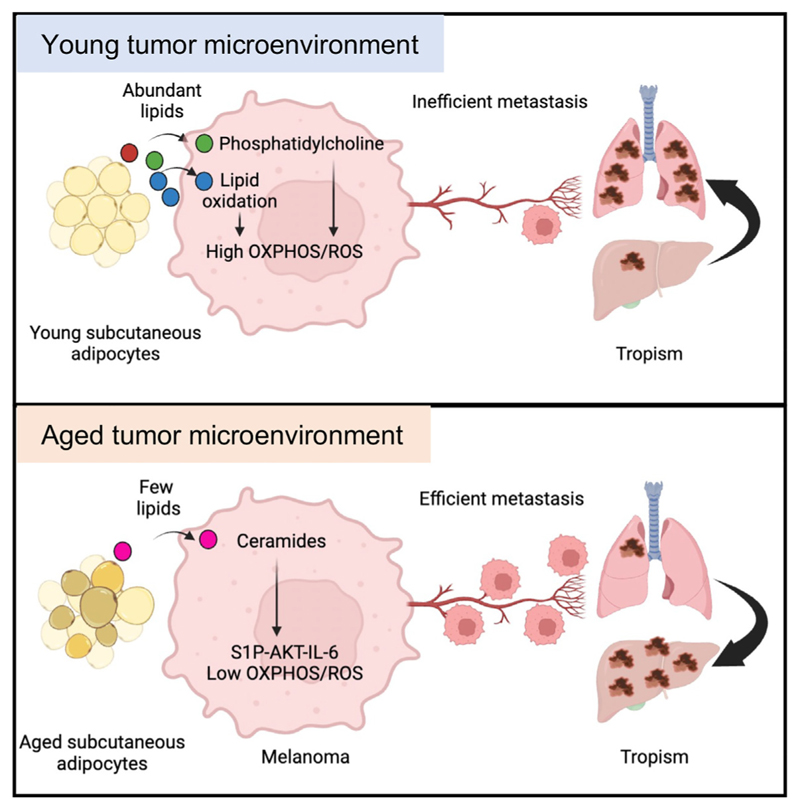

## Introduction

Malignant melanoma primarily follows two metastatic routes from the skin: via lymphatics to the lymph nodes and distant organs or by direct hematogenous spread to distant sites,^1^ where visceral disease is responsible for death. Both the lymphatic and hematogenous pathways convey a poor prognosis for patients once distant visceral disease is established.^2^ Cells in the primary melanoma can metastasize in parallel to different solid organ sites,^3,4^ indicating spread to organs from the primary is a key pathway to organ metastasis and death. Large studies recording the progression of melanoma metastasis show melanoma is more deadly in the aged population^5^ and aged patients have shorter disease-free survival.^1,5,6^ Lung is the most frequent solid organ of metastasis, followed by brain and liver.^7^ Intriguingly, brain metastases occur in younger patients, whereas liver metastases are more frequent in aged patients.^6,8^ The age-specific patterns of metastases are not understood, and the cues that drive melanoma to the lung, liver, or brain are not known.

Alterations in cancer cell metabolism are a hallmark of metastasis.^9^ High levels of oxidative stress in metastasizing melanoma cells limit hematogenous spread to visceral organs.^10–13^ In contrast, the lymphatic tumor microenvironment (TME) dampens oxidative stress, improving subsequent visceral seeding.^13^ Additionally, transcriptomic and metabolic cancer phenotypes and competencies are acquired after exposure to signals from the TME.^14–16^ However, the cues within the TME that impact the metastatic route, speed of disease and tropism are unknown. Cancer cells obtain nutrients from the TME and lipid uptake from the TME, as well as *de novo* lipid synthesis, sustain cell growth.^17,18^ In addition to their role as a source of energy, lipids and lipoproteins affect cell signaling,^18^ shaping cancer behavior and metastasis.^19–21^ Melanoma cells scavenge lipids from the TME for membrane and energy synthesis^22–24^ and fatty acid uptake by melanoma cells drives invasion and proliferation.^22,23^ Recent work shows that age significantly transforms the primary cutaneous TME and contributes to cancer progression,^24–27^ so we investigated whether primary TME lipid species vary by age and impact melanoma cell metabolism, intracellular signaling, hematogenous spread, and metastatic destiny.

## Results

### Aged human subcutaneous adipocytes contain and secrete fewer lipids

**Figure 1:**
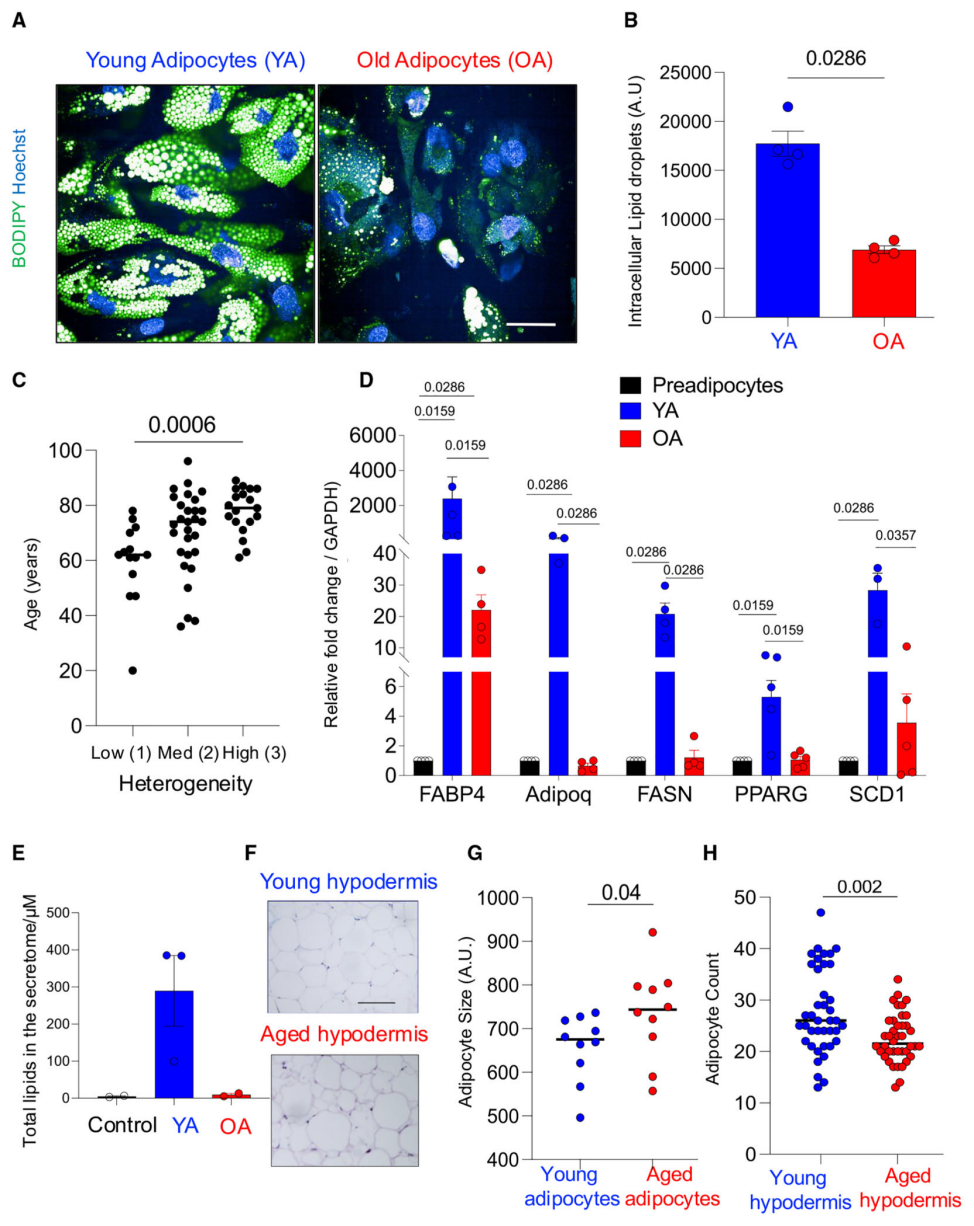
Aged human subcutaneous adipocytes contain and secrete fewer lipids (A) Photomicrographs of human subcutaneous adipocytes from old (>59 years) and young (<60 years) donors differentiated from preadipocytes. Intracellular lipids: Bodipy 493/503 (green), Hoechst (blue). Scale bar: 50μm. (B) Intracellular lipid droplet quantification, average intensity per cell young, old adipocytes; *n* = 4, two-sided Mann Whitney U test, *p* = 0.0286, AU: arbitrary units. Data represent mean and standard error. (C) Human healthy subcutaneous adipocyte diameter heterogeneity (low, medium and high) by age *n* = 62, Kruskal-Wallis test. Dots represent individual data, horizontal line represent mean. (D) Relative expression of adipocyte genes (*FABP4, ADIPOQ, PPARG, SCD1*, and *FASN*) in differentiated adipocytes, two-sided Mann Whitney U test, data represents mean and standard error, 4 (young), 5 (aged) biological replicates, and 2 technical repeats. *FABP4*, preadipocytes vs YA (*p* = 0.0159); *ADIPOQ*, preadipocytes vs YA (*p* = 0.0286); *PPARG*, preadipocytes vs YA (*p* = 0.0159); *SCD1*, preadipocytes vs YA(0.0286); *FASN*, preadipocytes vs YA (*p* = 0.0286); *FABP4*, preadipocytes vs OA (*p* = 0.0286); *ADIPOQ*, preadipocytes vs OA (*p* = 0.0286); *PPARG*, preadipocytes vs OA (*p* = 0.0286); *FABP4*, OA vs YA (*p* = 0.0159); *ADIPOQ*, OA vs YA (*p* = 0.0286); *PPARG*, OA vs YA (*p* = 0.0159); *SCD1*, OA vs YA(0.0357); *FASN*, OA vs YA (*p* = 0.0286). (E) Total lipid quantification in secretomes of young (YA, blue), old (OA, red) adipocytes, and serum free media (SFM control *n* = 3; old *n* = 2; young *n* = 3 biological replicates). Data represents mean with standard error. (F) H&E photomicrographs of hypodermis by age. Scale bar: 50 μm. (G) Adipocyte size, *n* = 6 random adipocytes measured per young (*n* = 10), old patient (*n* = 10), dots: average size/patient, line: median, AU: arbitrary units, two-way ANOVA, *p* = 0.04. (H) Adipocyte quantification in healthy abdominal hypodermis young (*n* = 10) and old (*n* = 10) patients with normal body mass index, *n* = 4 randomly selected 320 high power field counts per patient, two-sided Mann-Whitney U test, *p* = 0.002 line: median. See also Figure S1 and Tables S1 and S2.

Cutaneous adipocytes (adipocytes) are the main lipid reservoir in skin, secreting lipids and proteins that regulate epidermal and dermal functions including wound repair.^28^ The aged skin is functionally less competent,^29^ so we hypothesized lipids secreted by subcutaneous adipocytes decrease during the aging process. To test this hypothesis, we obtained subcutaneous preadipocytes from healthy human skin and differentiated them in culture to adipocytes (Table S1). Differentiated adipocytes from aged donors (median 76, range 60–91) contained fewer lipids and were prominently heterogeneous in their lipid content compared to young adipocytes (median 41, range 18–56, Figures 1A and 1B), a morphological feature in common with aged human hypodermis (Figure 1C). Furthermore, differentiated adipocytes from older donors expressed more SA-β-Gal and secreted higher levels of interleukin (IL)-6 than differentiated adipocytes from younger donors (Figures S1A–S1C); and these inflammatory markers in the aged, differentiated adipocytes were validated in aged human skin in the Genotype-Tissue Expression Project (GTEx)^30^ (Figure S1D).

Next, we compared the expression of adipocyte function and lineage genes in young and aged differentiated adipocytes (*FABP4, ADIPOQ, FASN, PPARG*, and *SCD*). RT-PCR of adipocyte genes revealed that aged adipocytes expressed lower levels of adipocyte genes (Figure 1D), and we confirmed that the adipocyte gene expression in human aged subcutis is significantly decreased compared to young subcutaneous tissue in the GTEx cohort^30^ (Figure S1E). Additionally, we analyzed the secretory component of young and aged adipocytes, which revealed aged adipocytes secrete fewer lipids to the extracellular space (Figure 1E; Table S2A). Mass spectrometry proteomic analysis of the secretome revealed old adipocytes secrete proteins associated with extracellular matrix turnover, such as matrix metalloproteinases, as well as proteins involved in the inflammatory response. In contrast, young adipocytes secrete proteins used for lipid transfer such as apolipoprotein E (Figures S1F and S1G; Table S2B). Finally, we investigated whether the human aged hypodermis compensates for the loss of adipocyte lipid gene expression and low lipid secretion by becoming hypercellular. For this, we compared the number and size of adipocytes in matched anatomic sites of young and aged skin of individuals of normal body weight, which revealed that the total cutaneous adipocyte count decreases with age (Figures 1F–1H). Taken together, these data indicate the human aged hypodermis contains fewer, larger adipocytes that express lower levels of adipocyte differentiation genes. Cutaneous preadipocytes obtained from human aged donors, which then differentiate in culture, replicate the less differentiated adipocyte phenotype and secrete fewer lipids compared to differentiated preadipocytes from young donors.

### Lipid uptake increases melanoma mitochondrial activity, OXPHOS, and reactive oxygen species

**Figure 2:**
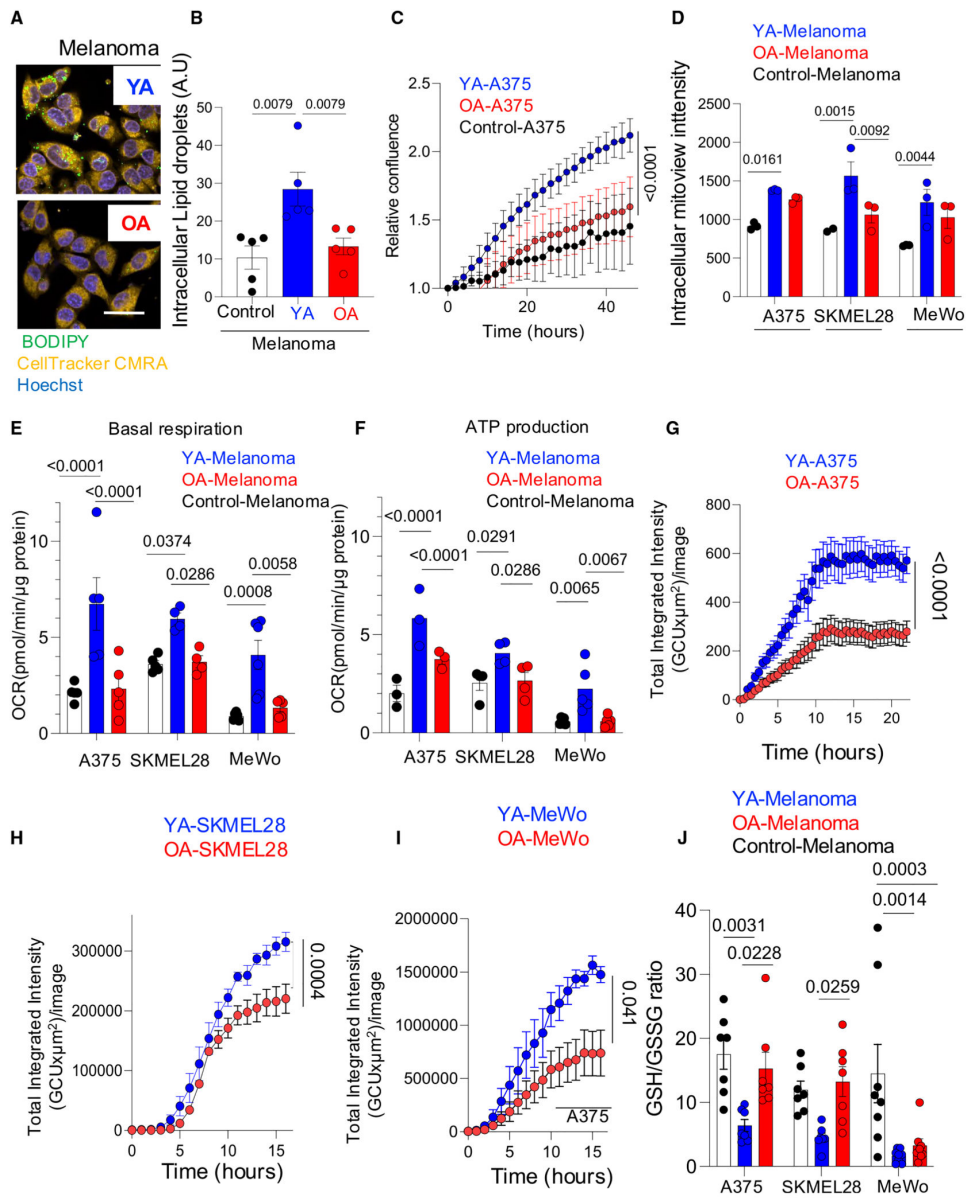
Lipid uptake increases mitochondrial activity, OXPHOS and ROS in melanoma cells (A) Lipid droplets in SKMEL28 cells after young (YA), old adipocyte (OA) secretome exposure. Blue: Hoechst, green: BODIPY, orange: CellTracker Orange CMRA. Scale bar: 50μm. (B) Intracellular lipid droplet quantification in cells after YA (blue), OA (red), or SFM control (white) exposure; *n* = 5 biological replicates, data represent mean and standard error, two-way ANOVA, AU: arbitrary units. SFM control vs YA (*p* = 0.0079); YA vs OA (*p* = 0.0079). (C) A375 cell proliferation after YA (blue), OA (red), and control (black) secretomes 48 h exposures, two-way ANOVA (control-A375 *n* = 3; YA-A375 *n* = 4, OA-A375 *n* = 4 biological replicates), data represents mean and standard error. (D) Active mitochondria in cells after YA (blue), OA (red) secretomes, or control (white), two-way ANOVA (control-A375 *n* = 3, YA-A375 *n* = 3, OA-A375 *n* = 3, control-SKMEL28 *n* = 2, YA-SKMEL28 *n* = 3, OA-SKMEL28 *n* = 3, control-MeWo *n* = 3, YA-MeWo *n* = 3, OA-MeWo *n* = 3 replicates), data represents mean and standard error. Control-A375 vs YA-A375, *p* = 0.0161; Control-SKMEL28 vs YA-SKMEL28, *p* = 0.0015; Control-MeWo vs YA-MeWo, *p* = 0.0044. (E) Mitochondrial basal respiration oxygen consumption rate (OCR) in melanoma cells after YA (blue), OA (red), or control (white), two-way ANOVA (control-A375 *n* = 5, YA-A375 *n* = 5, OA-A375 *n* = 5, control-SKMEL28 *n* = 5, YA-SKMEL28 *n* = 4, OA-SKMEL28 *n* = 4, control-MeWo *n* = 6, YA-MeWo *n* = 6, OA-MeWo *n* = 6 biological replicates), data represents mean and standard error. Control-A375 vs YA-A375, *p*≥ 0.0001; Control-SKMEL28 vs YA-SKMEL28, *p* = 0.0374; Control-MeWo vs YA-MeWo, *p* = 0.0008; YA-A375 vs OA-A375, *p* ≥ 0.0001; YA-SKMEL28 vs OA-SKMEL28, *p* = 0.0286; YA-MeWo vs OA-MeWo, *p* = 0.0058. (F) ATP production in melanoma cells after YA (blue), OA (red), or control (white), two-way ANOVA (control-A375 *n* = 3, YA-A375 *n* = 3, OA-A375 *n* = 3, control-SKMEL28 *n* = 4, YA-SKMEL28 *n* = 4, OA-SKMEL28 *n* = 4, control-MeWo *n* = 5, YA-MeWo *n* = 5, OA-MeWo *n* = 5 biological replicates), data represents mean and standard error. Control-A375 vs YA-A375, *p*% 0.0001; Control-SKMEL28 vs YA-SKMEL28, *p* = 0.02981; Control-MeWo vs YA-MeWo, *p* = 0.0065; YA-A375 vs OA-A375, *p*% 0.0001; YA-SKMEL28 vs OA-SKMEL28, *p* = 0.0286; YA-MeWo vs OA-MeWo, *p* = 0.0067. (G–I) ROS levels normalized to cell confluence in (G) A375, (H) SKMEL28, and (I) MeWO melanoma cells after YA (blue), OA (red) 24 h (*n* = 3 in G and *n* = 4 in H and I) data represents mean and standard error, two-sided Mann-Whitney U test. (J) Ratio of reduced (GSH) and oxidized (GSSG) glutathione in melanoma cells after YA (blue), OA (red), or control (white), two-way ANOVA (control-A375 *n* = 7, YA-A375 *n* = 7, OA-A375 *n* = 7; control-SKMEL28 *n* = 7, YA-SKMEL28 *n* = 7, OA-SKMEL28 *n* = 7, control-MeWo *n* = 8, YA-MeWo *n* = 8, OA-MeWo *n* = 8 replicates). All data are mean and standard error. Biological replicates refer to adipocyte secretomes. Control-A375 vs YA-A375, *p* = 0.0031; Control-MeWo vs YA-MeWo, *p* = 0.0003; YA-A375 vs OA-A375, *p* = 0.0228; YA-SKMEL28 vs OA-SKMEL28, *p* = 0.0259; YA-MeWo vs OA-MeWo, *p* = 0.0014. See also Figure S2.

We investigated whether lipids secreted by young (YA) and old (OA) cutaneous adipocytes impact melanoma cell behavior. Human melanoma cell lines (A375, SKMEL28, and MeWo) were exposed to YA and OA donor secretomes (YA-A375, OA-A375, YA-SKMEL28, OA-SKMEL28, YA-MeWo, and OA-MeWo). YA secretomes contain more lipids (Figure 1E), and consequently, we found exposure to YA led to greater lipid uptake by melanoma cells (Figures 2A and 2B), and increased melanoma cell proliferation compared to OA secretome exposure (Figures 2C, S2A, and S2B). We next investigated the metabolic consequences of lipid uptake in melanoma cells, and found YA exposure increased mitochondrial activity, oxidative phosphorylation (OXPHOS) and ATP production in melanoma cells to a greater extent than OA-exposed melanoma cells. This increase in OXPHOS and ATP production after YA was independent of total mitochondrial mass^31^ and independent of mitochondrial transfer to melanoma cells (Figures 2D–2F, S2C, and S2D). Furthermore, YA-exposed melanoma cells (YA-melanoma) generated high levels of reactive oxygen species (ROS) and had a decreased glutathione (GSH) to oxidized glutathione (GSSG) ratio (GSH/GSSG (Figures 2D–2J and S2E–S2G), in keeping with a higher mitochondrial respiration and redox stress after lipid oxidation. In contrast, OA-exposed melanoma cells (OA-melanoma) had lower mitochondrial activity and respiration, lower ROS, higher GSH/GSSG ratio compared to YA-melanoma; and relied on glycolysis as an energy source (Figures 2D–2J and S2E–S2H).

To verify lipids in YA secretomes lead to increased OXPHOS, we delipidated YA and OA secretomes with Cleanascite, before transferring the secretome to melanoma cells. This revealed that lipid-stripped YA and OA secretomes did not increase melanoma mitochondrial activity, OXPHOS, ROS, or affect the GSH/GSSG ratio (Figures S2I–S2O. Furthermore, the irreversible inhibition of the fatty acyl chain transfer to the intermembrane space of the mitochondria (CPT1a) with etomoxir (Figures S2P and S2Q), or CPT1a lentiviral knockdown targeting both isoforms of CPT1a in YA-melanoma significantly reduced melanoma proliferation, OXPHOS, and ATP production (Figures S2R–S2V). These data confirm lipids from young and aged adipocytes, taken up by melanoma cells, are used to drive melanoma proliferation and increase OXPHOS and ROS. The pharmacological inhibition or genetic ablation of lipid metabolism in melanoma cells inhibits these effects. Young adipocyte lipids increase proliferation, OXPHOS, and ROS to a greater extent than aged adipocyte lipids.

### The aged lipid environment promotes metastasis

Successfully metastasizing melanoma cells must adapt to oxidative stress as cells enter the bloodstream and decreasing ROS with antioxidants in circulating melanoma cells promotes metastasis.^10,12^ These data raise the possibility that melanoma cells exposed to YA, leading to increased OXPHOS, ROS, and oxidative stress, are less prone to metastasize than melanoma cells exposed to OA, which generate fewer ROS and consequently have a higher GSH/GSSG ratio. To test this *in vivo* in immunocompetent animals, we injected murine melanoma cells (5555, YUMM1.7) into the aged and young adipocyte microenvironment of C57BL/6 young and aged mice. We chose the adipocyte-rich mammary fat pad (MFP) because the mouse hypodermis, in contrast to human hypodermis, is too thin to envelop tumor growth; and additionally, because MFP adipocytes are embryologically part of the subcutaneous adipocyte tissue.^32^ We validated the MFP is a relevant skin adipocyte model, showing the aged MFP expresses and produces lower levels of key adipogenic proteins (CD36, FABP4, FASN, and PPARG) compared to young MFP (Figures S3A and S3B; Table S3); and old murine adipocytes express genes linked to adipocytokine inflammation, in contrast to young tissue, which expressed lipoproteins (Figure S1F; Table S3). Additionally, the expression of FABP4, a critical adipogenic gene, is decreased in the aged MFP and the aged murine hypodermis, compared to young tissue (Figure S3C). Thus, the RNA and proteome of the MFP replicate the hallmarks of subcutaneous hypodermis, and critically, the aging process in the MFP shares hallmarks of human hypodermis aging.

**Figure 3:**
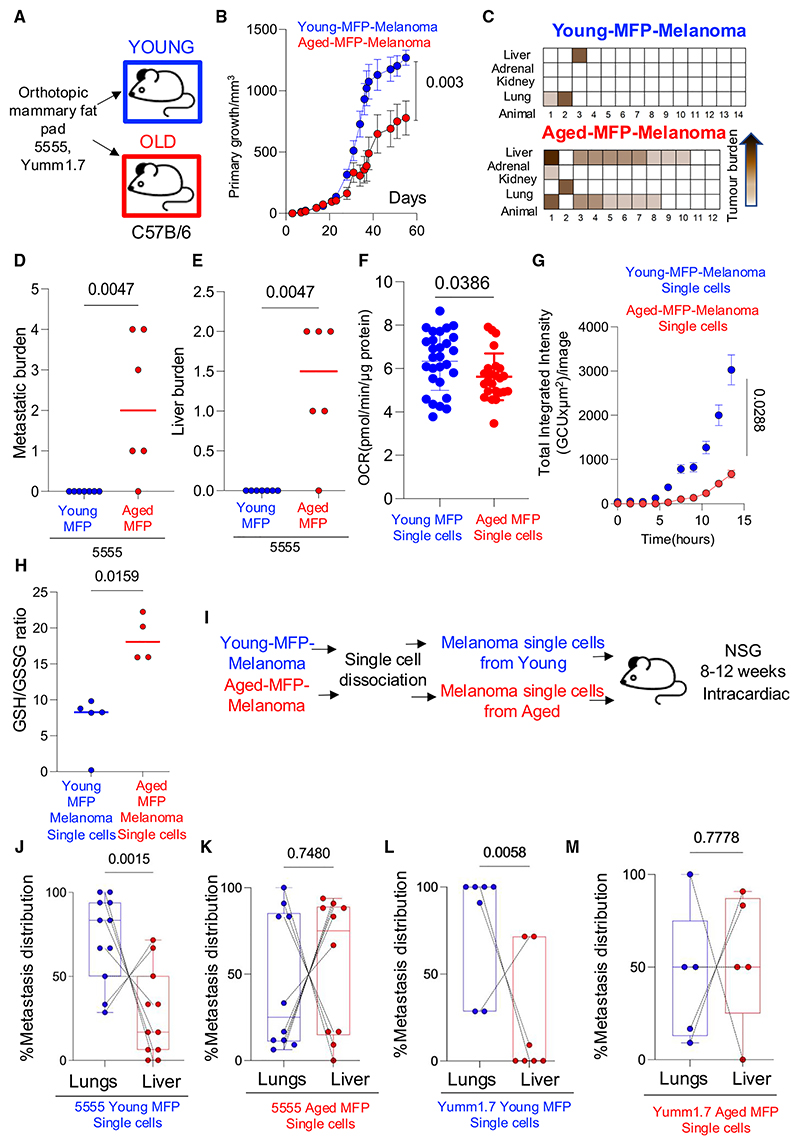
The aged lipid environment promotes metastasis (A) Experimental model for orthotopic mammary fat pad (MFP) injection of melanoma cells. (B) Primary tumor growth in MFP of 5555 and Yumm1.7 melanoma cells in young (8–12 weeks, *n* = 12, blue) and aged (12–18 months, *n* = 12, red) C57B/6 animals. Young-MFP-melanomas: *n* = 8 5555; *n* = 4 Yumm1.7; aged-MFP-melanomas: *n* = 8 5555; *n* = 4 Yumm1.7. Mean volume and standard error, two-sided Mann-Whitney U test, *p* = 0.003. (C) Metastatic burden in lungs, liver, kidneys, adrenal glands in young-MFP-melanomas and aged-MFP-melanomas. Range: 50% tumor replacement of organ (dark brown) to <5% tumor replacement (light brown). (D) Bar graph of metastatic burden 5555 aged-MFP-melanoma (red, *n* = 6) and 5555 young-MFP-melanoma (blue, *n* = 7). Data points: sum of metastatic burden in all organs of individual animals, lines: median, two-sided Mann-Whitney U test, *p* = 0.0047. (E) Bar graph of liver burden aged-MFP-5555 (red, *n* = 6), 5555 young-MFP-5555 (blue, *n* = 7). Points: liver burden, lines: medians, two-sided Mann-Whitney U test, *p* = 0.0047. (F–H) (F) Seahorse basal respiration, 5 technical replicates, *p* = 0.0386, (G) intracellular ROS, *p* = 0.0286, and (H) GSH/GSSG ratio of single melanoma cells dissociated from young-MFP-5555 and aged-MFP-5555. Respiration *n* = 4 young, *n* = 3 aged, ROS *n* = 5 young, *n* = 3 aged, GSH/GSSG *n* = 5 young, *n* = 4 aged biological replicates; lines: mean and standard error, two-sided Mann-Whitney U test, *p* = 0.0159. (I) Experimental model for intracardiac injection of MFP melanoma cells. (J–M) Paired wise graph metastatic distribution in lungs (blue) and liver (red) of (J) young-MFP-5555 (*n* = 11), *p* = 0.0015, (K) aged-MFP-5555 (*n* = 10), *p* = 0.7480, (L) young-MFP-Yumm1.7 (*n* = 7), *p* = 0.0058, and (M) aged-MFP-Yumm1.7 (*n* = 5), *p* = 0.778 single dissociated melanoma cells. Dots are lung and liver metastasis for each mouse, dotted line matches lung and liver of each animal, whisker graph is median and quartile distribution, two-sided Mann-Whitney U test. Metastasis distribution: organ tumor burden divided by the total tumor burden (lungs and liver) 3100. See also Figures S3 and S4 and Table S3.

Therefore, we injected syngeneic 5555 or YUMM1.7 mouse melanoma cells orthotopically into the MFP, which revealed that melanomas developing in the adipocyte-rich MFP of young, immunocompetent C57BL/6 mice (young-MFP-melanoma) grew more rapidly than melanoma cells injected in the aged MFP (aged-MFP-melanoma, Figures 3A, 3B, S4A, and S4B), in keeping with enhanced proliferation due to greater lipid availability in the young adipocyte environment. The MFP injection model, in contrast to the subcutaneous model, allows visceral metastases to develop, so we next compared the metastatic burden of young-MFP-melanomas to aged-MFP-melanomas. Whole body autopsies revealed animals with aged-MFP-melanomas had a significantly higher burden of organ metastases than animals with young-MFP-melanomas (Mann-Whitney U test, *p* = 0.0047, Figures 3C, 3D, and S4C). Interestingly, aged-MFP-melanomas were more metastatic to the liver than young-MFP-melanomas (Mann-Whitney U test, *p* = 0.0047, Figures 3E and S4D). These data support that high oxidative stress decreases hematogenous spread, as young-MFP-melanomas have fewer metastases.

The higher rate of metastasis of aged-MFP-melanomas could be due to both local TME factors, leading to lower oxidative stress and improved metastatic fitness, or due to systemic aging factors such as weakened immunity. To investigate whether local or systemic age factors contribute to the higher metastatic rate of aged-MFP-melanomas, we dissociated single cells from the young-MFP-melanomas and aged-MFP-melanomas. Single cells harvested from young-MFP-melanomas retained higher OXPHOS, ROS, and lower GSH/GSSG ratio than single dissociated cells from aged-MFP-melanomas (Figures 3F–3H). Next, we injected dissociated tumor cells from young-MFP-melanomas or aged-MFP-melanomas into the left ventricles of immunocompromised young (8–12-week-old) animals and compared the rate of metastasis (Figure 3I). Strikingly, cells harvested from aged-MFP-melanomas, injected into young animals, retained a higher metastatic efficiency compared to cells harvested from young-MFP-melanomas injected into young animals (5555, Yumm1.7; Figure S4E). Intriguingly, mice injected with melanoma cells from young-MFP tumors more frequently seeded the lung (Figures 3J and 3L). This was validated using different batches of tumour cells isolated from multiple young-MFP-melanomas. Conversely, melanoma cells harvested from aged-MFP-melanomas, injected into the ventricles of young mice, preferentially seeded the liver (Figures 3K and 3M). We validated this using an additional model of tail vein cancer dissemination, which confirmed Yumm1.7 cells harvested from young-MFP-melanomas preferentially seeded the lung, in contrast to Yumm1.7 cells from aged-MFP-melanomas that established large liver metastases (Figures S4F–S4H). Thus, exposure to an aged, adipocyte-rich environment enhances metastasis and promotes liver tropism, whereas the young adipocyte environment decreases total metastases and decreases liver tropism.

### Stromal lipid cues impose a gradient of OXPHOS, leading to distinct tropism

**Figure 4:**
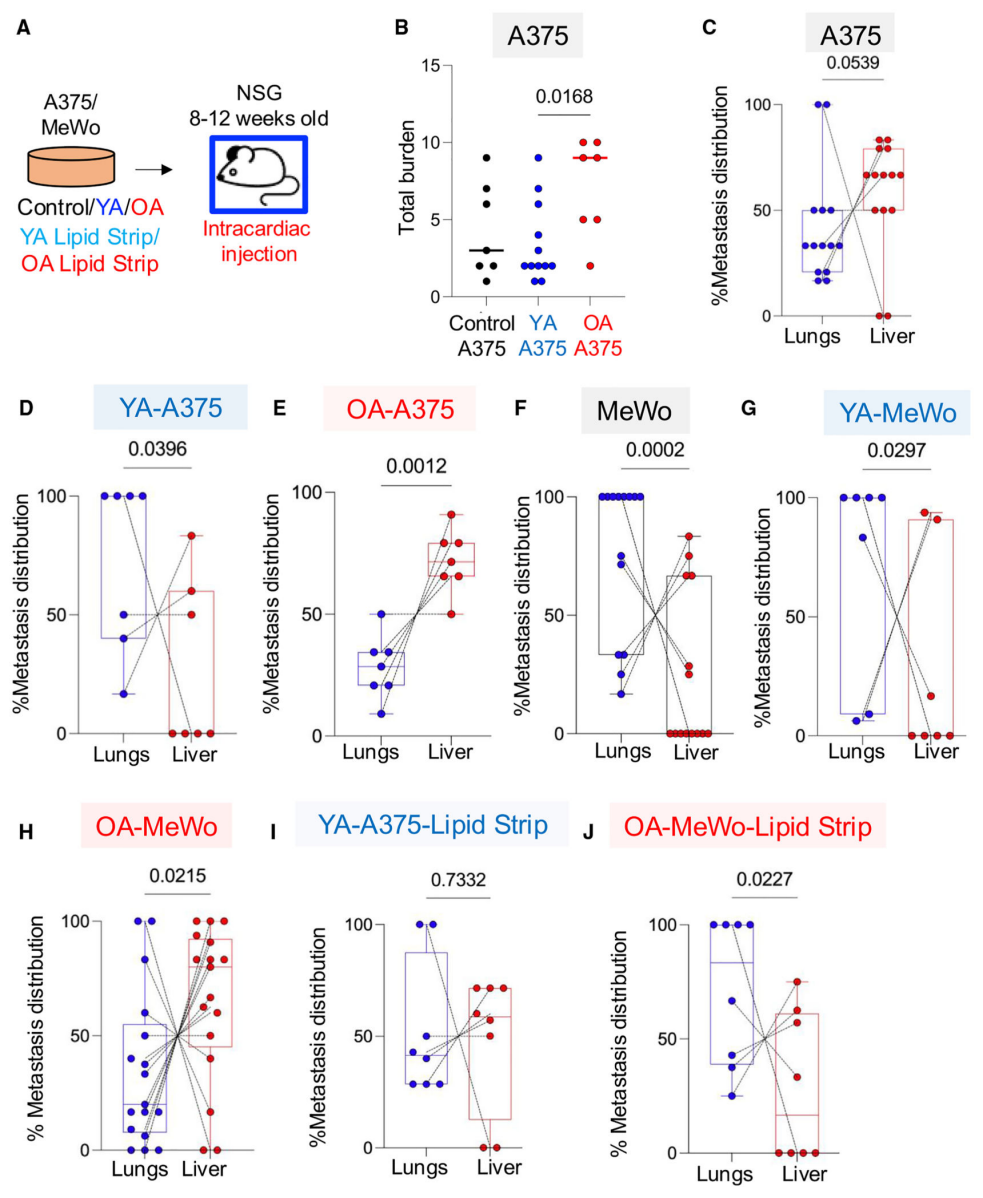
Lipid availability dictates metastatic tropism (A) Experimental model of intracardiac inject of melanoma cell lines treated *in vitro* with adipocyte secretomes. (B) Graph of total metastasis burden after A375 (black, *n* = 7), YA-A375 (blue, *n* = 12), OA-A375 (red, *n* = 7) injection, data points represent individual mice and line represents median, *p* = 0.0168. (C–J) Paired wise whisker plot metastasis distribution of lungs (blue) and liver (red) in mice injected with (C) A375 (*n* = 14), *p* = 0.0539, (D) YA-A375 (*n* = 7), *p* = 0.0396, (E) OA-A375 (*n* = 7), *p* = 0.0012, (F) MeWo (*n* = 14), *p* = 0.0002, (G) YA-MeWo (*n* = 7), *p* = 0.0297, (H) OA-MeWo (*n* = 17), *p* = 0.0215, (I) YA-A375-lipid strip (*n* = 8), *p* = 0.7332 and (J) OA-MeWo-lipid strip (*n* = 8), p = 0.0227. Dots: metastatic distribution lung and liver, dotted lines match lung and liver for each animal, whisker graph median and quartile metastatic distribution, two-sided Mann-Whitney U test. See also Figure S4.

To extend our analysis to human melanoma, we exposed A375 human cells to YA (YA-A375) or OA secretomes (OA-A375), and injected A375, YA-A375, and OA-A375 cells into the hearts of immunocompromised 8–12-week-old NSG mice. A375 cells have an inherent greater tropism to the liver than the lung of NSG mice, but not the brain.^21^ We found OA-A375 cells were more metastatic than control A375 cells (Figures 4A–4E), and furthermore, YA-A375 cells were significantly less tropic to the liver and more likely to colonize the lung than A375 and OA-A375 cells (Figures 4A–4E). Thus, young lipid exposure led to higher melanoma cell ROS/OXPHOS, reduced rate of liver metastasis in favor of lung colonization. In contrast, aged lipid exposure enhanced metastatic burden primarily to the liver, in immunocompetent (C57BL/6) and immunocompromised (NSG) mice, in murine and human cells. To further explore liver tropism after aged lipid exposure, we used human melanoma MeWo cells, which are inherently more tropic to the lung than the liver.^21^ We injected MeWo, YA-MeWo, and OA-MeWo cells into 8–12-week-old NSG animals and found YA exposure did not increase metastatic burden or change the natural tropism of MeWo cells to the lung (Figure 4F). However, OA-MeWo cells were significantly more metastatic and liver tropic than MeWo and YA-MeWo cells (Figures 4F and 4H).

To confirm the metastatic efficiency and tropism are due to lipids and not due to other secreted components, we stripped the lipids from YA secretomes and exposed A375 melanoma cells to the lipid-stripped-YA (YA-A375-lipid-stripped), which confirmed that YA-A375-lipid-stripped cells lost their YA-induced lung tropism, reverting to enhanced liver tropism (Figures 4D and 4I). Similarly, OA-MeWo-lipid-stripped cells lost their liver tropism and reverted to lung tropism (Figures 4H and 4J). Furthermore, young-MFP-melanomas expressed higher levels of *FABP4, CPT1a*, and *CD36* than aged-MFP-melanomas (Figure S4I) validating that the young-MFP is an environment that enhances lipid metabolism in melanoma cells. Thus, we find that YA and OA lipids at primary sites regulate lipid metabolism and OXPHOS in melanoma cells; and dictate metastatic burden and tropism. Aged lipid exposure drives liver metastasis.

### Phosphatidylcholine secreted by young stromal adipocytes increases PI3K-AKT signaling and OXPHOS, shifting tropism to the lung

**Figure 5:**
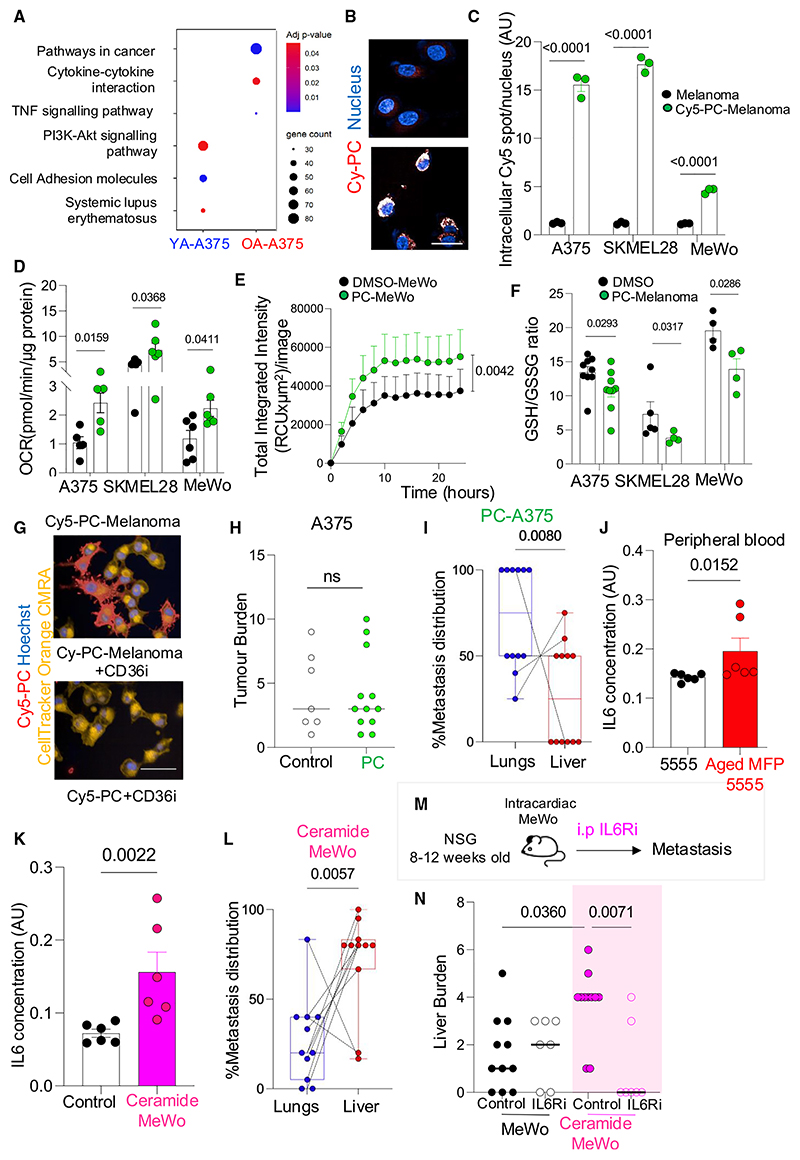
Phosphatidylcholine drives lung tropism, ceramide drives liver tropism (A) Pathways enriched for overexpressed genes YA-A375 (left) and OA-A375 (right). Sphere size: number of enriched genes in pathway, color: significance (log_10_FDR, red most significant). (B) Representative images A375 treated with SFM and 1 μM Cy5-PC. Blue: Hoechst, red: Cy5-PC. Scale bar: 50 μM. (C) Intracellular Cy5-PC per nuclei in melanoma A375, SKMEL28, and MeWo after 1 μM of Cy5-PC or DMSO, data mean and standard error, *n* = 3, two-way ANOVA, *p* < 0.0001. (D) Mitochondrial basal respiration OCR A375, SKMEL28, and MeWo after treatment with 5 μM PC (green) or DMSO (black), data mean OCR with standard error, two-way ANOVA (DMSO-A375 *n* = 5, PC-A375 *n* = 5; control-SKMEL28 *n* = 6, PC-SKMEL28 *n* = 6, control-Mewo *n* = 6, and PC-MeWo *n* = 6 replicates). DMSO-A375 vs PC-A375, *p* = 0.0159; DMSO-SKMEL28 vs PC-SKMEL28, *p* = 0.0368; DMSO-MeWo vs PC-MeWo, *p* = 0.0411. (E) Mitochondrial ROS in MeWo cells after 5 μM PC (green) or DMSO 24 h, *n* = 3, data mean and standard error, two-sided Mann-Whitney U test, p = 0.0042. (F) GSH/GSSG ratio in cells after 5 μM PC (green) or DMSO (black). Data mean and standard error, two-sided Mann-Whitney U test (DMSO-A375 *n* = 9, PC-A375 *n* = 9, DMSO-SKMEL28 *n* = 5, PC-SKMEL28 *n* = 4, DMSO-MeWo *n* = 4, and PC-MeWo *n* = 4 biological replicates). DMSO-A375 vs PC-A375, *p* = 0.0293; DMSO-SKMEL28 vs PC-SKMEL28, *p* = 0.0317; DMSO-MeWo vs PC-MeWo, *p* = 0.0286. (G) Representative images SKMEL28 after 1 μM Cy5-PC (top) and 1 μM Cy5-PC and salvianolic acid B (CD36i-25 μM, bottom), DMSO 6 h; blue: Hoechst, red: 1 μM Cy5-PC, orange: CellTracker Orange CMRA. Scale bar: 50 μM. (H) Total tumor burden in mice injected with A375 (black, *n* = 7), PC-A375 (green, *n* = 12) cells, data points represent individual mice and line represents median. (I) Paired wise whisker plot of metastasis distribution lungs (blue) liver (red) in mice injected with PC-A375 (green, *n* = 12). Dots represent metastatic distribution, dotted lines match the corresponding lung and liver scores for each animal, whisker graph median and quartile metastatic distribution, two-sided Mann-Whitney U test, *p* = 0.0080. (J) IL-6 blood concentration of C57BL/6 8–12-week-old mice injected with either 5555 (*n* = 6, black) or aged-MFP 5555 cells (*n* = 6, red), data represents mean and standard error, *p* = 0.0152. (K) IL-6 concentration in conditioned media of MeWo cells after exposure to DMSO-MeWo (*n* = 6, black) or ceramide-MeWo 100 nM (*n* = 6, magenta), data represents mean and standard error, *p* = 0.0022. (L) Paired wise whisker plot of metastasis distribution lungs (blue) and liver (red) in mice injected with Ceramide-MeWo (*n* = 11). Dots: metastatic distribution for each mouse, dotted lines match lung and liver for each animal, whisker graph median and quartile metastatic distribution, two-sided Mann-Whitney U test, *p* = 0.0057. (M) IL-6 receptor inhibitor I(L6R)i experimental model. (N) Liver metastasis with MeWo (*n* = 11, black), ceramide-MeWo (*n* = 11, magenta), and MeWo cells treated with IL6Ri (*n* = 7) injected into animals, data points represent individual mice and line represents mean, Kruskal-Wallis test Control- vs IL6Ri, *p* = 0.0360; Ceramide vs Ceramide Il6Ri, *p* = 0.0071. See also Figures S4–S6 and Table S4.

Lipid species can act as intracellular signaling molecules, so we explored whether lipid species in the adipocyte secretomes modify intracellular signaling, metabolism, metastasis, and tropism when they are taken up by melanoma cells. For this, we compared the transcriptome of A375, YA-A375, and OA-A375 cells, which revealed YA exposure strongly upregulates genes of the PI3K pathway (Figure 5A; Table S4A), and we validated these transcriptional effects in YA-MeWo cells (Figure S4J; Table S4B).

Intracellular lipids contribute to tumor cell adaptation and metastasis^19–21^; and the phospholipid lysophosphatidic acid drives mitogenic signaling via the PI3K-AKT pathway in pancreatic cancer.^33,34^ Therefore, we investigated whether phospholipids in YA drive PI3K-AKT signaling in YA-melanoma cells. For this, we first confirmed phospho-AKT^S473^ is upregulated in young-MFP-melanomas compared to aged-MFP-melanomas (Figures S4K and S4L), then used mass spectrometry lipidomic analyses. Lipidomics analyses revealed that old adipocytes secrete fewer total lipids than young adipocytes. Importantly, we observed the composition of lipid species varied by age. While phosphatidylcholines (PCs) were proportionately increased in the young adipocyte secretome (Figure S4M), aged secretomes proportionately contain more ceramides than young adipocyte secretomes (Table S2). Importantly, we found melanoma cells grown in the young-MFP environment had a higher PC content than melanoma cells in the aged-MFP (Figure S4N), and furthermore, young-MFP tissue contained more PC than aged-MFP tissue (Figure S4O). Additionally, lipid species in the MFP vary by age (Table S5). Finally, melanoma cells have a higher PC content after YA secretome exposure compared to melanoma cells exposed to OA (Figure S4P). Taken together, these data show PC is a hallmark of the young lipid milieu.

Next, we tested whether PC upregulates PI3K and impacts metastasis and tropism. For this, we confirmed that tracked, soluble PC (Cy5-PC) is taken up by melanoma cells (Figures 5B and 5C). Then, we exposed melanoma cells to a physiologically relevant dose of synthetic PC (PC-A375, PC-SKMEl28, and PC-MeWo; 5 μM), which revealed PC-melanoma cells, like YA-melanoma cells, increased phospho-AKT^S473^ (Figures S4Q–S4U), melanoma cell proliferation, OXPHOS, and ROS production and reduced GSH/GSSG (Figures 5D–5F and S5A–S5F).

Fatty acids can enter cancer cells from the extracellular space via the CD36 receptor,^20^ so we tested whether CD36 mediates PC entry into melanoma cells. For this, we tracked PC uptake in melanoma cells treated with a CD36 inhibitor (salvianolic acid B, CD36i), which confirmed that inhibition of CD36 abrogates Cy5-PC uptake (Figures 5G and S5G), melanoma proliferation and ROS production (Figures S5A–S5F).

Next, we investigated whether PC uptake regulates proliferation, OXPHOS, and ROS via PI3K and pmTORC2 signaling. PC-melanoma and YA-melanoma cells were treated with either a pharmacological inhibitor of AKT (LY294002) or a pMTORC2 inhibitor (JR-AB2-011); which confirmed inhibition of PI3K (AKT and pMTORC2) significantly reduced proliferation, OXPHOS, and ROS in YA-melanoma and PC-melanoma cells (Figures S5H–S5P). Taken together, these results show PC is more abundant in the young TME, is taken up via CD36 by melanoma cells where it drives PI3K via pmTORC2, OXPHOS, and ROS. Blocking fatty acid metabolism,^19^ PC uptake via CD36 or PI3K signaling, target lipid signaling after YA lipid exposure.

To study how PI3K drives OXPHOS, we investigated whether pAKT^S473^ activates mitochondrial respiration. Imaging analyses revealed pAKT^S473^ transferred from the cytoplasm to the mitochondria (Figure S5Q) and western blot confirmed that pAKT^S473^ increased in the mitochondria following PC and YA exposure (Figure S6A). Previous studies show that phosphorylated AKT increases pro-survival BCL-2 proteins,^35–37^ and BCL-2 can stimulate mitochondrial respiration in tumor cells.^38,39^ Therefore, we explored whether cells exposed to YA or PC upregulate BCL-2 and pro-survival pathways, and confirmed that YA, but not OA exposure, increase BCL-2 in melanoma cells (Figure S6B), and furthermore, RNA sequencing (RNA-seq) confirms YA and PC, but not OA exposure, upregulate antiapoptotic genes (Table S4). To test whether BCL-2 is linked to the respiratory phenotype after lipid exposure, we treated PC-melanoma and YA-melanoma cells with the BCL-2 inhibitor ABT199,^40^ which significantly reduced OXPHOS (Figure S6C), indicating BCL-2 mediates OXPHOS after lipid and PC exposure. Finally, we studied whether PC, which increases OXPHOS, limits metastatic spread and liver tropism *in vivo*, and found PC-A375 cells were less efficient at establishing liver metastases in NSG mice (Figures 5H and 5I). These results highlight that stromal PC, taken up by melanoma cells, activates PI3K signaling. pAKT^S473^ localizes to the melanoma cell mitochondria where it increases OXPHOS via BCL-2.

### Ceramides secreted by aged stromal adipocytes promote liver metastasis

Next, we investigated whether lipid species in OA secretomes impact metastasis and liver tropism via intracellular signaling. Mass spectrometry lipidomics analyses revealed that although OA secrete fewer total lipids (Figure 1E), they secrete proportionately more ceramide species than young adipocytes (Figure S6D; Table S2A). Additionally, the aged-MFP had a higher content of some ceramide species (Figure S6E; Table S7). Aged tissues accumulate ceramide species^41^ and ceramides are sphingolipids that convert to sphingosine-1-phosphate (S1P). S1P signaling drives cancer metastasis by activating STAT3 and IL-6 production.^42^ Therefore, we tested whether ceramides in the aged TME drive metastatic spread and liver tropism via the S1P-STAT3-IL-6 signaling axis, and confirmed that SPHK1 expression, a critical gene in the S1P pathway, was increased in OA-melanoma cells and aged-MFP-melanomas compared to YA-melanoma and young-MFP-melanomas (Figures S6F and S6G; Table S4). Furthermore, melanoma STAT3 activation (pSTAT3^Tyr70^) and IL-6 secretion following exposure to ceramides was significantly increased (Figures 5J, 5K, S6H, and S6I). To validate that the aged lipid subcutaneous environment impacts melanoma cell metabolic programs in human skin by age, we compared the activation of the S1P pathway and PC catabolism in single cell RNA-seq of subcutaneous melanoma metastases of young (*n* = 2) and aged patients^43^ (*n* = 2). This confirmed that the S1P and glycolysis signatures were increased in melanoma cells in the aged subcutaneous tissue (Figure S6J), whereas PC catabolism and phosphatidylinositol signaling were upregulated in melanoma cells embedded in the young hypodermis (Figures S6K and S6L).

We next tested whether OA exposure impacts systemic IL-6 and found higher IL-6 levels in the peripheral blood of C57BL/6 animals injected with melanoma cells from aged-MFP-melanomas (Figure 5J). Furthermore, melanoma MeWo cells exposed to physiological levels of ceramides (ceramide-MeWo) increased IL-6 secretion and shifted metastatic tropism to the liver (Figures 4G, 4H, and 5L). Critically, treatment with an IL-6R inhibitor (100 μg/dose, total 5 doses) significantly decreased liver metastases of ceramide-MeWo cells (Figures 5M and 5N), which indicates that stromal ceramide promotes melanoma liver metastasis via IL-6.

### ROS levels imposed by stromal lipid cues dictate tropism

**Figure 6:**
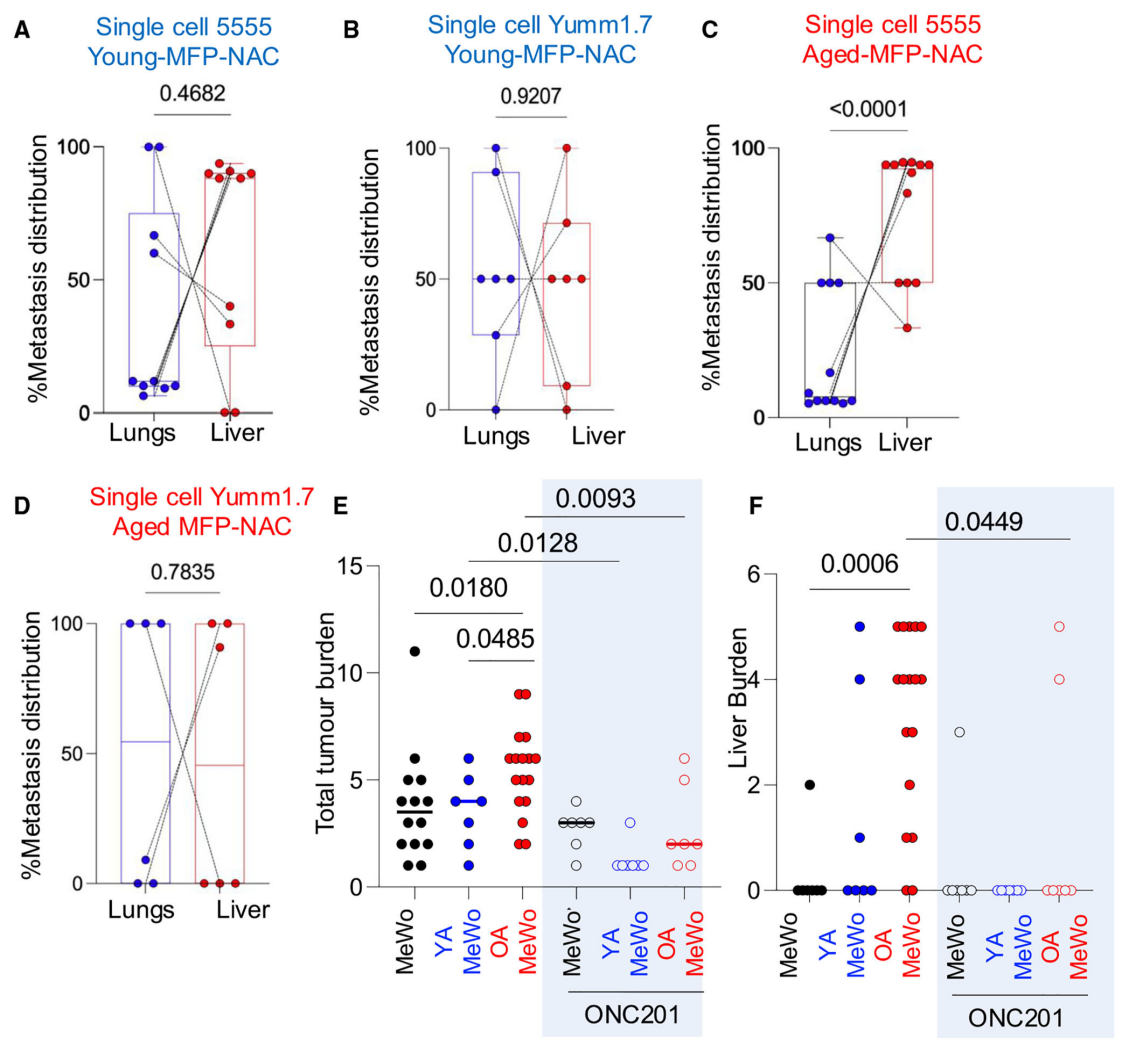
Dietary antioxidants and inhibition of OXPHOS regulate tropism and metastatic capacity (A–D) Paired wise whisker plot of lung (blue) and liver (red) metastasis after single dissociated (A) young-MFP-5555 cells, *p* = 0.4682, (B) young-MFP-Yumm1.7 cells, *p* = 0.9207; (C) aged-MFP-5555 cells, *p* < 0.0001, and (D) aged-MFP-Yumm1.7 cell injection, with NAC (200 mg/kg/day) supplementation. Dots: lung and liver metastasis, dotted lines match lung and liver for each animal, whisker graph median and quartile metastatic distribution, two-sided Mann-Whitney U test (young-MFP-5555 *n* = 10, aged-MFP-5555 *n* = 7, young-MFP-Yumm1.7 *n* = 12, and aged-MFP-Yumm1.7 *n* = 6). (E and F) (E) Total tumor burden MeWo vs OA-MeWo, *p* = 0.0180; YA-MeWo vs OA-MeWo, *p* = 0.0485; YA-MeWo vs YA-MeWo-ONC201, *p* = 0.0128; OA-MeWo vs OA-MeWo-ONC201, *p* = 0.0093 and (F) liver burden in mice injected with MeWo (black, *n* = 14), YA-MeWo (blue, *n* = 7), OA-MeWo (red, *n* = 17), MeWo + ONC201(10 mg/kg/day) (white and black outline, *n* = 7), YA-MeWo +ONC201(white and blue outline, *n* = 7), and OA-MeWo +ONC201(white and red outline, *n* = 7), lines: median, two-sided Mann-Whitney U test. MeWo vs OA-MeWo, *p* = 0.0006; OA-MeWo vs OA-MeWo-ONC201, *p* = 0.0449. See also Figure S7.

We found stromal lipid uptake by melanoma cells sets the metabolic rate of OXPHOS, so we investigated the role of OXPHOS in tropism. Low lipid uptake after OA exposure, leading to moderate OXPHOS, strongly enhances metastasis; whereas higher OXPHOS after YA exposure limits metastatic potential. Therefore, we hypothesized that supplementation with the antioxidant (AOX) N-acetyl-cysteine (NAC) would increase the metastatic burden and liver tropism specifically in YA-melanoma cells. To test this, we injected melanoma cells (5555, Yumm1.7), from young-MFP-melanomas or aged-MFP-melanomas into age-matched, immunocompromised 8–12-week-old NSG animals supplemented with NAC (200 mg/kg/day). This revealed that young-MFP-melanoma cells in NAC-treated animals were metastatic to the liver compared to non-supplemented animals, which retained lung tropism (Figures 6A–6D, S7A, and S7B). As expected, NAC supplementation did not significantly change the high metastatic efficiency or liver tropism of aged-MFP-melanoma cells, which have baseline lower OXPHOS levels (Figures 6A–6D). These data indicate that ROS levels impact metastatic efficiency and tropism.

Because moderate levels of OXPHOS (after OA exposure) enhance metastasis, we next tested whether OXPHOS is necessary for metastasis. For this, we inhibited OXPHOS with ONC201, an imipridone small molecule inhibitor of dopamine receptor D2 and caseinolytic peptidase, which activates mitochondrial proteolysis and powerfully impairs OXPHOS.^44^ We found that ONC201 (3 μM) decreased proliferation, reduced OXPHOS, ATP production, and maximal respiration of YA-melanoma and OA-melanoma cells (Figures S7C–S7G). ONC201 behaved in a similar manner to rotenone, a complex I inhibitor, which reduces mitochondrial respiration in melanoma cells (Figure S7H). *In vivo*, ONC201 treatment (10 mg/kg/day) powerfully reduced the metastatic potential of MeWo, OA-MeWo, and YA-MeWo cells (Figure 6E), showing that melanoma cells rely on OXPHOS for visceral spread. Specifically, ONC201 more powerfully inhibited YA-MeWo metastasis, which have a greater metabolic reliance on OXPHOS than OA-MeWo cells; and significantly decreased liver tropism of OA-MeWo (Figures 6E and 6F). Importantly, untreated MeWo cells retained their metastatic capacity (Figure 6E), indicating ONC201 specifically targets melanoma cells after exposure to TME cues. Thus, stromal lipids impose varying levels of OXPHOS, which enables and determines the rate of metastasis. Aged lipids induce moderate OXPHOS and enhance metastasis and liver tropism, and young lipids impose high OXPHOS, which decreases metastasis and drives tropism to the lung.

### Distinct metastatic trajectories to brain and liver

**Figure 7:**
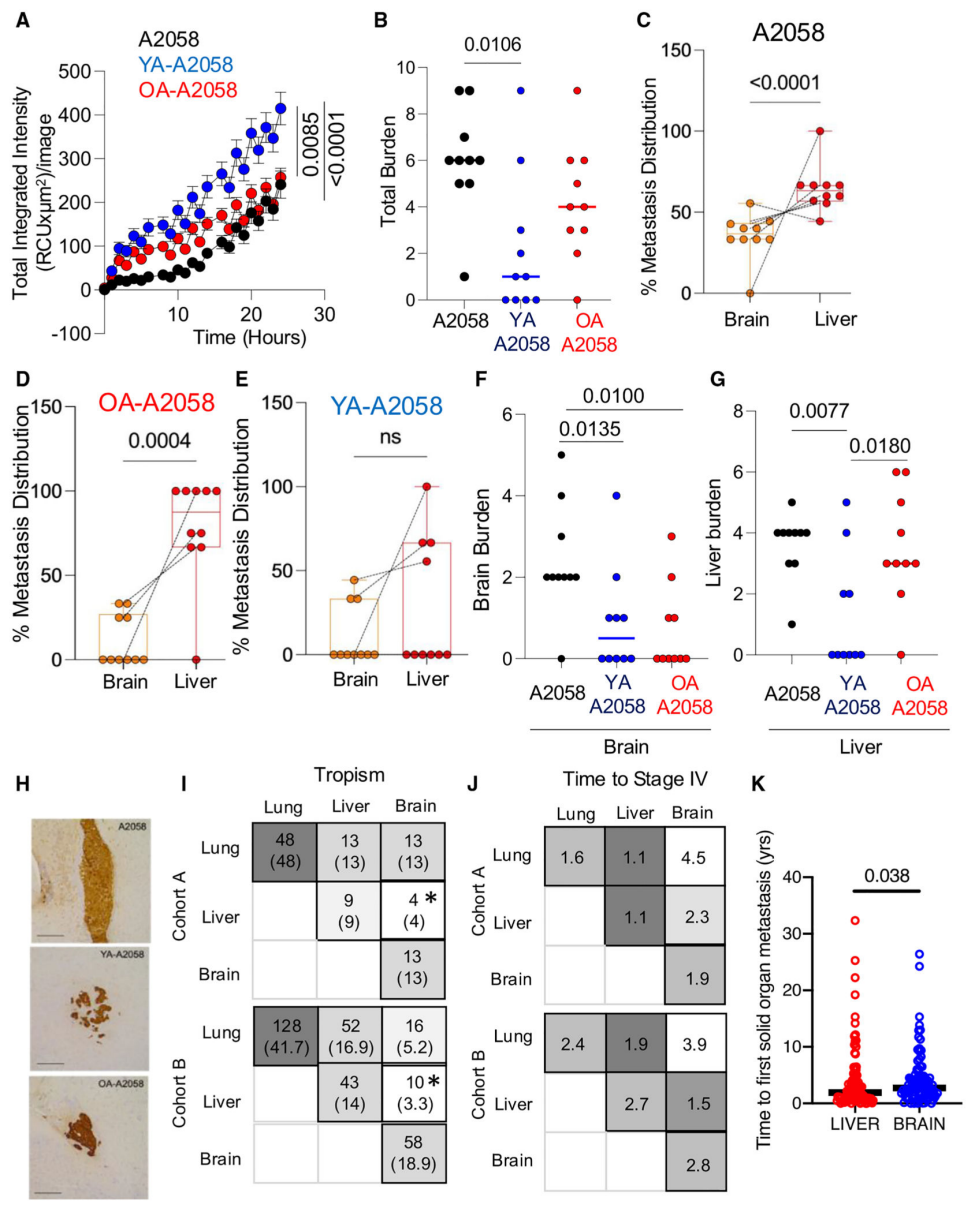
Distinct melanoma metastatic trajectories to brain and liver (A) Mitochondrial ROS in A2058 cells with SFM control-A2058 (*n* = 4, black), YA-A2058 (*n* = 4, blue), and OA-A2058 (*n* = 4, red) 24 h, data represents mean and standard error, one-way ANOVA. Control-A2058 vs YA-A2058, *p* < 0.0001; YA-A2058 vs OA-A2058, *p* = 0.0085. (B) Total metastatic burden control-A2058 (*n* = 10, black), YA-A2058 (*n* = 10, blue), and OA-A2058 (*n* = 10, red), line represents median score, two-sided Mann-Whitney U test. Control-A2058 vs YA-A2058, *p* = 0.0106. (C–E) Paired wise whisker plot metastasis distribution of brain (orange) liver (red) of (C) A2058 (*n* = 10 mice), *p* < 0.0001, (D) OA-A2058 (*n* = 10), *p* = 0.0004, and (E) YA-A2058 (*n* = 10) cells, dot score per mouse and line represents median, two sided Mann-Whitney U test. (F and G) (F) Graph of brain tumor burden. Control -A2058 vs OA-A2058, *p* = 0.0100; Control-A2058 vs YA-A2058, *p* = 0.0135. (G) liver tumor burden, control-A2058 (*n* = 10, black), YA-A2058 (*n* = 10, blue), and OA-A2058 (*n* = 10, red). Line represents median score, two-sided Mann-Whitney U test. Control-A2058 vs YA-A2058, *p* = 0.0077; YA-A2058 vs OA-A2058, *p* = 0.0180. (H) Photomicrograph of anti-GFP immunohistochemistry metastasis control-A2058 (top), YA-A2058 (middle), and OA-A2058 (bottom). Scale bar: 200 μm. (I) Stage IV melanoma patients first site of visceral metastases. Number and proportion of patients with one and two organ involvement in brain, lung, and liver as first solid organ metastasis in two cohorts. Asterisks show Z *p* value. (J) Time from primary to stage IV diagnosis in patients with one and two organ involvement in brain, lung, and liver. (K) Time to first solid organ metastasis to liver versus brain (*n* = 112 liver, *n* = 95 brain, Mann-Whitney U paired test, data points represent individual patients, line represents mean), *p* = 0.038. See also Table S6.

We next studied whether stromal lipid exposure and OXPHOS/ROS levels affect the rate of brain metastatic dissemination. We used human A2058 melanoma cells, which have dual tropism primarily to the liver and the brain,^21^ exposing them to YA (YA-A2058) and OA secretomes (OA-A2058). In keeping with our previous data, we found YA-A2058 cells had higher ROS levels than OA-A2058 and A2058 cells (Figure 7A), lower metastatic capacity overall (Figure 7B), and reduced liver colonization (Figures 7C–7G). In contrast, OA-A2058 cells had lower brain tropism but retained the metastatic ability to colonize the liver *in vivo* (Figures 7B–7G). A2058, YA-A2058, and OA-A2058 animals were sacrificed at the same time, and we noted that YA-A2058 and OA-A2058 presented micro metastatic brain disease compared to A2058, which had large, multifocal brain metastases (Figure 7H). This indicates that after lipid exposure, which drives OXPHOS, the metastatic process is less efficient to the brain, and brain metastases develop at a slower pace. However, OA-A2058 cells retain high liver metastatic efficiency, suggesting that liver spread, not brain metastases, are more likely to afflict the aged earlier during disease progression. These data strongly support that liver and brain metastases follow metabolically distinct programs of metastasis.

### Distinct spatial and time metastatic trajectories in humans

The time and spatial pattern of metastatic progress can vary dramatically between patients; from rapid, synchronous disease affecting multiple organs leading to early death, to a protracted clinical course over decades. Our *in vivo* models indicate that metastatic efficiency and tropism are driven by metabolic programs that are imprinted by stromal lipids. Lipid components of the young TME increase melanoma OXPHOS, leading to inefficient metastatic spread, and at a slower pace, to the brain. In contrast, the lipids in the aged TME lead to moderate OXPHOS and increase total and liver metastasis. Brain metastases are more common in younger patients, and conversely, older patients have more total metastases, more liver metastases, and a worse outcome.^6,8^ To further explore the relevance of our findings in humans, we audited the patterns and time to metastasis of two independent cohorts of stage IV melanomas, classified by their first sites of visceral metastasis to the most common organs: lung, liver, and brain. Single metastases to the lung were confirmed the most common, suggesting lung is the most permissive tissue for melanoma colonization. Next, we confirmed brain and liver are the second most common visceral metastatic sites; and critically, we found liver and brain metastases were the least likely metastatic events to co-occur (Figure 7I; Table S6). We next compared the time from diagnosis to first metastasis to the brain and liver, which confirmed that liver metastases develop more rapidly than brain metastases (Figures 7J and 7K; Table S6, liver median 1.918 years, *n* = 111; brain median 2.745 years, *n* = 95; *p* value = 0.0384). Thus, metastatic efficiency to brain and liver differs in humans, and our experimental data show lipid cues in the stroma, which differ by age, dictate the pattern of spread. Metastatic organ destiny is non-random, with distinct spatial and time patterns governed by metabolic programs. The inverse correlation between liver and brain metastases, with different time to develop, strongly support our findings of opposing metastatic programs governing liver and brain spread.

## Discussion

Visceral adipocytes contribute to cancer progression,^45–47^ and we show that cutaneous adipocytes contribute to melanoma by releasing lipids as a source of oxidation and biomass production to fuel growth and metastasis, and also by providing signaling lipid species to melanoma cells. Lipids secreted by skin adipocytes impose transcriptomic and metabolic OXPHOS in melanoma cells, and OXPHOS dictates metastatic potential and tropism. Following lipid uptake in young skin, melanoma cells increase lipid signaling and shift their transcriptome and metabolism to adapt to lipid availability. Lipids from cutaneous adipocytes promote melanoma proliferation, and melanomas in a lipidrich, young environment uptake triglycerides, fatty acids, and complex lipids for consumption and growth. PC and fatty acid uptake from the young TME fuel lipid oxidation and melanoma proliferation. Oxidation generates high ROS and oxidative stress, which limits melanoma metastatic spread. Conversely, melanomas in a lipid-poor, aged skin environment take up fewer lipids and have moderate OXPHOS metabolism. Lower oxidative stress promotes melanoma metastasis. Thus, we found that *in vivo* ROS, imposed by stromal lipid oxidation, dictates metastatic tropism in immunocompetent and immunocompromised animals. High OXPHOS/ROS reduces liver metastasis and favors lung colonization. In contrast, moderate, lower OXPHOS facilitates metastasis and strong liver tropism. Dietary supplementation with antioxidants decreases oxidative stress, which promotes liver tropism in the young TME.

Melanoma cells exposed to more lipids in the young TME can metastasize to the brain. However, this process is inefficient and occurs at a slow pace. In contrast, melanoma cells with moderate OXPHOS imposed by the aged lipid environment rapidly form liver metastases and lead to animal death. Thus, it is possible that our model of moderate OXPHOS, leading to rapid liver metastases, does not allow sufficient time for melanoma cells to conquer the brain TME, as the animals succumb of liver disease early. Thus, these preliminary brain data suggest that brain metastases arise in younger animals, at a slow pace, from cells that survive high levels of oxidative stress imposed by the primary TME lipids and have no liver tropism. Importantly, human stage IV patients with brain metastases are younger^6,8,48,49^ and we show human and mouse brain metastases have a longer latency. In contrast, metastases to the liver are more common in aged patients,^6^ and our human data show liver metastases have a shorter time to progression. Critically, our clinical audit and other cohorts^50^ show brain and liver metastases are inversely correlated, which strongly supports that liver and brain disease arise from opposing metastatic programs.

Young patients who are exposed to lipid rich TMEs frequently present lymphatic spread, and the lymph TME reduces oxidative stress and improves melanoma cell survival.^13^ Our work fits this paradigm, revealing the aged, lipid-poor skin imposes lower oxidative stress on melanoma cells and improves solid organ metastasis.

Phosphatidylcholine from young adipocytes, taken up by melanoma cells activates the PI3K-AKT signaling pathway, pAKT translocation to the mitochondria, lipid oxidation and increases OXPHOS, leading to lung metastases. Blocking fatty acid uptake via the CD36 receptor inhibits melanoma metastasis^20,51^; and we show CD36 inhibition additionally blocks PC uptake, PI3K activation and OXPHOS, leading to fewer metastases. Critically, recent studies link lipid metabolism downstream of PI3K to brain tropism in breast cancer.^21^

In contrast, ceramides from aged adipocytes activate S1P-STAT3-IL-6, IL-6 secretion, and liver tropism. IL-6 production in pancreatic cancer promotes the formation of a premetastatic niche in the liver by recruiting myeloid cells and increasing liver fibrosis.^33^ We show that high IL-6 after ceramide exposure drives liver tropism in immunocompromised animals. Furthermore, other cell types, including aged dermal fibroblasts contribute ceramides to the extracellular space.^24^ Thus, it is possible that the depletion of lipids coupled to an increase in ceramides that characterizes aged skin also occurs in other tissues and contributes to aged cancer progression in other cancers. Mutations accumulate and expand clonally in aged healthy tissues, affecting tissue renewal and function.^52^ Our work raises the possibility that decreasing lipid provision in the aging stroma may enable malignant progression of transformed clones.

Multiple spatial transcriptomic and oxidative metabolic states coexist in primary melanoma, leading to distinct metastatic potentials.^12^ Cell heterogeneity leads to a range of metastatic potentials and tropism within a single tumor, increasing the complexity of metastasis. Our data suggest that lung metastases, which are the most frequent, likely occur from a wide dynamic range of OXPHOS. Moderate OXPHOS drives liver disease, and melanoma cells with higher OXPHOS that survive the metastatic process can seed the lung and brain, but not the liver.

OXPHOS is a limiting factor of metastasis that reflects agedependent lipid availability. This work adds to the growing evidence that extrinsic cues from the microenvironment shape tumor cell behavior, and tumor behavior is dynamic. Our work identifies lipid availability, lipid uptake, lipid oxidation, PI3K pathway, ceramide pathway, and OXPHOS inhibition as potential adjuvant therapeutic targets to delay melanoma hematogenous spread to vital organs, and these therapeutic strategies should be trialed by patient age or stromal lipid availability.

### Limitations of the study

Our study exposed melanoma cells to adipocytes, but it is likely that lipid ratios are more complex in multicellular human skin and add further pressures on melanoma cells. Similarly, we used homogeneous cancer lines, whereas metabolic tumor cell heterogeneity will additionally impact the metastatic process *in vivo* in cells under nutrient competition. Given that melanoma is a highly heterogeneous cancer, whether our findings apply to all clinical and molecular subtypes remains to be established. Finally, further work is necessary to validate and identify specific pathways regulated by lipids in melanoma and other cancer cells that are organ tropic specific.

### Star★Methods

#### Key Resources Table

**Table:** 

REAGENT or RESOURCE	SOURCE	IDENTIFIER
Antibodies		
Phospho-Akt (Ser473)(D9E)XP Rabbit mAb	Cell Signaling	Cat #4060; RRID: AB_2315049
Phospho-Akt (Thr308)(244F)Rabbit mAb	Cell Signaling	Cat #4056; RRID:AB_331163
Akt Antibody	Cell Signaling	Cat #9272; RRID:AB_329827
Phospho-mTOR (Ser2448)(D9C2)XP Rabbit mAb	Cell Signaling	Cat#5536; RRID:AB_10691552
mTOR Antibody	Cell Signaling	Cat#2972; RRID:AB_330978
Phospho-Stat3 (Tyr705) Antibody	Cell Signaling	Cat#9131; RRID:AB_331586
STAT3 Antibody	Cell Signaling	Cat#9132; RRID:AB_331588
BCL2 (124) Mouse mAb	Cell Signaling	Cat#15071; RRID:AB_2744528
Anti- beta Tubulin Antibody	Abcam	ab6046; RRID:AB_2210370
Anti- Cytochrome C Antibody [7H8.2C12]	Abcam	ab13575; RRID:AB_300470
CPT1A Antibody	Proteintech	Cat#66039-1; RRID:AB_11041710
Anti-TOMM20 Antibody [4F3]-BSA and Azide Free	Abcam	ab56783; RRID:AB_945896
Anti-Rabbit IgG, HRP-linked Antibody	Cell Signaling	Cat#7074; RRID:AB_2099233
Anti-Mouse IgG, HRP-linked Antibody	Cell Signaling	Cat#7076; RRID:AB_330924
Goat anti- Mouse Alexa Fluor 488	Thermo Fisher	A32723; RRID:AB_2633275
Goat anti-Rabbit Alexa Fluor 555	Thermo Fisher	A32732; RRID:AB_2633281
Fatty Acid Synthase (C20G5) Rabbit mAb	Cell Signaling	Cat#3180S; RRID:N/A
CD36 (E8B7S)Rabbit mAb	Cell Signaling	Cat#28109S; RRID:N/A
PPAR*γ* (C26H12) Rabbit mAb	Cell Signaling	Cat #2435s; RRID:N/A
Anti- FABP4 Antibody [EPR3579]	Abcam	ab92501; RRID:AB_10562486
*InVivo* MAb anti-mouse IL-6R	Bio X cell	BE0047; RRID:AB_1107588
Biological samples		
Human Dog ears for Preadipocytes’ isolation	This study	Biobank
Chemicals, peptides, and recombinant proteins		
Trypsin -EDTA (0.25%), phenol red	Gibco	25200056
DMEM with glutamax	Thermo Fisher	10566016
FBS	Thermo Fisher	A5256801
Penicillin - Streptomycin	Thermo Fisher	15140122
Glutamax	Thermo Fisher	35050061
Non essential Amino acids	Thermo Fisher	11140050
Cleanscite Lipid Removal Kit	Biotech Support Group	X2555-100
Dispase II	Gibco	Cat no: 17105041
Collagenase I	Gibco	Cat no: 17018029
Adipocytes Maintenance media	Zenbio	AM-1
PGM-2 Preadipocyte Growth Medium -2 Bullekit	Lonza	PT 8002
BODIPY 493/503	Cayman	CAS 121207-31-6
DMSO	Sigma Aldrich	D540
PBS	Thermo Scientific	J61196.AP
Cy5 -PC	Avanti Lipids	850483
Hoechst	Thermo Fisher	62249
CellTracker Orange CMRA	Invitrogen	C34551
Mitoview 633	Biotium	BT70055
MitoTracker Green Dyes	Invitrogen	M7514
Seahorse XF base medium	Agilent	103335-100
Glucose Solution	Gibco	A2494001
Sodium Pyruvate (100mM)	Gibco	11360070
Triton X 100	Sigma Aldrich	X100-500ML
Sodium chloride	Sigma Aldrich	S6546
Tris	Sigma Aldrich	93362
MOPS	Sigma Aldrich	M1254
Chloroform	Thermo Fisher	J67241.K2
Tween 20	Sigma Aldrich	P1379
10X Tris Buffered Saline (TBS)	Bio-Rad	1706435
Methanol	Merck	1.06007
Acetone	Sigma Aldrich	320110
UltraPure SDS Solution,10%	Thermo Fisher	15553027
4% Paraformaldehyde in PBS	Thermo Scientific	J61899.AK
10%Formalin	Sigma Aldrich	HT501640-19L
Tris- Glycine- SDS Buffer 10x Concentrate	Sigma Aldrich	T7777-1L
Sucrose	Sigma Aldrich	S9378
Ethylene -bis (oxyethylenenitrilo)tetraacetic acid (EGTA)	Sigma Aldrich	ED2SS
Beta- mercaptoethanol	Sigma Aldrich	M6250-M6250
Laemmli Buffer	Bio-Rad	610747
Phosphatase Inhibitor	Roche	4906845001
Protease Inhibitor	Roche	11836153001
Mini-PROTEAN TGX gels	Bio-Rad	4568084
Precision Plus Ladder	Bio-Rad	1610374
1XTGS buffer	Bio-Rad	1610732
Ponceau Stain	G-Biosciences	786-575
TransBlot Tubro system	Bio-Rad	170-4270
BSA	Sigma	A9418
ECL western blot substrate	Promega	W1001
Polybrene	Santa Cruz Biotechnology	sc-134220
Puromycin	Sigma Aldrich	P8833-10MG
Egg-PC	Avanti Polar Lipids	840051
Ceramide	ThermoFisher	D-7540
JR-AB2-011	MedChem Express	HY-122022
LY294002	Cambridge Bioscience	70920-5mg-CAY
ABT199	ApeXBio	A8194
Salvianolic acid	Cayman chemical	9001577
Etomoxir	MedChemExpress	HY-50202
Rotenone	Sigma Aldrich	R8875
Trizol solution	Invitrogen	12044977
Antimycin A	Sigma Aldrich	A8674
Carbonyl cyanide 4-(trifluoromethoxy)phenylhydrazone	Sigma Aldrich	C920-10MG
Oligomycin	Sigma Aldrich	75351-5MG
SYBR Green	Sigma Aldrich	4913850001
CellROX Green	Thermo Fisher	C10444
MitoSOX Red mitochondrial superoxide indicator	Thermo Fisher	M36008
Collagenase IV	Worthington Biochemical	LS004186
ONC201	Apex Bio	A8724
N-acetylcysteine	Sigma Aldrich	A0737
Critical commercial assays		
Seahorse XF Cell Mito Stress Test kit	Agilent	103015-100
Seahorse XF Cell Energy Phenotype Test kit	Agilent	103325-100
Pierce BCA Protein Assay Kit	Thermo Fisher	23225
Senescence detection kit	Abcam	ab65351
Qiagen Omniscript reverse transcriptase kit	Qiagen	205113
Qiagen RNA extraction kit	Qiagen	74104
Promega GSG/GSSG-Glo Assay Kit	Promega	V6611
Phosphatidylcholine Assay Kit	Abcam	ab83377
Murine IL6 ELISA	R&D systems	SM6000B
Human IL6 ELISA	R&D systems	DY206
Deposited data		
Lipidomics of human Young and Aged Adipocyte secretome	This study	Table S2
Proteomics of human Young and Aged adipocyte secretome	This study	Table S2
RNA sequencing of murine young and aged mammary fat pad (processed data)	This study	Table S3
RNA sequencing of murine young and aged mammary fat pad (raw data)	This study	GEO: GSE292629
RNA sequencing of A375 exposed to secretome (processed data)	This study	Table S4
RNA sequencing of A375 exposed to secretome (raw data)	This study	GEO: GSE292627
RNA sequencing of MeWo exposed to secretome (processed data)	This study	Table S4
RNA sequencing of MeWo exposed to secretome (raw data)	This study	GEO: GSE292628
Lipidomics of murine young and aged mammary fat pad	This study	Table S5
Single cell sequencing of subcutaneous metastases	Pozniak et al., 2024^43^	KU Leuven RDR: https://doi.org/10.48804/GSAXBN
Experimental models: Cell lines		
5555	Gifted by Prof. Richard Marais	
Yumm 1.7 (male murine)	ATCC	CRL-3362; RRID: CVCL_JK16
A2058-GFP (male human)	Gifted by Prof. Vicky Sanz Moreno and Laura Stennett	N/A
A375 (female human)	ATCC	CRL-1619; RRID: CVCL_0132
SKMEL28 (male human)	ATCC	HTB-72; RRID: CVCL_0526
MeWo (male human)	ATCC	HTB-65;RRID:CVCL_0445
Subcutaneous Preadipocytes	Lonza	PT 5020
Experimental models: Organisms/strains		
Mouse: NSG	Charles River	N/A
Mouse: C57BL/6N Crl	Charles River	N/A
Oligonucleotides		
qPCR primers: Supplemental files Table S9		
Recombinant DNA		
shRNA lentiviral particles A	Santa Cruz Biotechnology	sc-108080
CPT1a shRNA (h) lentiviral particles	Santa Cruz Biotechnology	sc-40376, shRNA
Software and algorithms		
ImageJ 1.52as	Fiji	
GraphPad Prism Ver. 8.2.0	GraphPad Software, Inc.	
Harmony	Perkin Elmer	
Microsoft Excel	64-bit, Microsoft	
Seurat package (version 4.0.2).	Bioconductor	
DeSEQ2	Bioconductor	
GFold (v1.1.4)	Bioconductor	
Incucyte Software	Essen Bioscience, 2020C Rev1	
HALO imaging	Indica Labs, New Mexico USA	
Other		
Tungsten carbide beads	Qiagen	69989

### Experimental Model And Study Participant Details

#### Human metastatic cohorts

All patients from Aix-Marseille University Hospital and Institut Valencia Oncologia who presented metastatic disease to either brain, lung or liver were audited, from clinical multidisciplinary team melanoma lists, from the past 10 years. We examined the metastatic time to visceral metastasis and the first site of visceral metastasis. All patients consented at both institutions for research and audit purposes with their local ethics committees.

#### Human samples

A prospective cohort of patient preadipocyte cultures was established from redundant skin acquired during surgical resection of the wide local excision of skin cancer patients treated at the tertiary referral cancer Christie NHS hospital. Ethical approval to establish cell lines was granted by the local Biobank committee (17_AMVI_01), which required signed informed consent from all participants.

#### Mice

All procedures involving animals were performed under the Home Office approved project license P8ADED6C8 and PP0466403, and UK Home Office regulations under the Animals (Scientific Procedures) Act 1986. The study received ethical approval by the Cancer Research UK Manchester Institute’s Animal Welfare and Ethics Review Body (AWERB). All mice were maintained in pathogen-free, ventilated cages in the Biological Resources Unit at our Institute, and allowed free access to irradiated food and autoclaved water in a 12 h light/dark cycle, with room temperature at 21 ± 2°C. All cages contained wood shavings, bedding, and a cardboard tube for environmental enrichment. All female mice were obtained from Charles River and acclimatized in the breeding resource unit for a week prior to the experiment. Mice weighing less than 16g were not used for the experiment. Animals were randomized to different groups. C57B/6 strain J and NSG animals were used.

#### Cell culture

All melanoma cell lines (A375, MeWo, SKMEL28, A2058-GFP, 5555, Yumm1.7) were cultured under standard conditions in DMEM (Thermo fisher) supplemented with 10% Fetal Bovine Serum (Life technologies) and 1% Penicillin and Streptomycin. A2058-GFP cell lines were provided by Laura Stennett, Gilbert Fruhwirth^,^ and Victoria Sanz-Moreno. Yumm1.7 was further supplemented with 5% essential amino acids (Thermo fisher). All cells were incubated in 5% CO_2_ except for A2058-GFP (10% CO_2_). Cell line identity was confirmed by STR and mycoplasma testing undertaken before each *in vivo* experiment, monthly testing for *in vitro*.

### Method Details

#### Human sample processing

The samples were transported with an ice pack and kept cold. Immediately upon arrival, the surgical redundant skin from donors (surgical dog ears) were cut vertically and stored at -150C with matched blood. Subcutaneous fat was scraped off the skin layer and minced into small pieces using a scalpel. The minced fat was placed in a Lo protein bind Eppendorf tube (Eppendorff Cat no:22431102) for further processing. The rest of the skin sample (epidermis and dermis) was placed in Dispase II solution (Gibco®Cat no: 17105041) (25U/ml) overnight so that epidermis and dermis could be separated. Upon separation of epidermis and dermis, a part of both the layers was used for culture. The dermis and hypodermis were finely minced and digested in Collagenase I (Gibco® Cat no: 17018029) at 580U/ml. The hypodermis is digested for 2 hours, and the dermal layer digested for 6 hours. Preadipocytes from the hypodermis were separated by straining the solution using a 100μm strainer. The flow-through was centrifuged at 300x g for 10 minutes and the pellet re-suspended in 20% FCS supplemented DMEM/F12. For dermis, the digested material was strained using a 70um strainer and the flow through centrifuged at 300xg for 10 minutes. The pellet was then re-suspended in 20% FCS supplemented DMEM and plated in a six well plate. Gender, age and other information are described in Table S1.

#### Differentiation of adipocytes

Preadipocytes from donors (including Christie Biobank or Lonza, PT5020) were differentiated into adipocytes using preadipocyte basal medium (Lonza, PT 8002) supplemented with recombinant human insulin, dexamethasone, indomethacin and IBMX according to manufacturer protocol. Morphological changes in adipocytes were observed within a week of differentiation and incubation with differentiation medium for 20 days resulted in 70% differentiation of preadipocytes to adipocytes with lipid accumulation. Differentiated adipocytes were then maintained in subcutaneous adipocyte maintenance medium (Zenbio, AM-1) until further use.

#### Mammary fat pad injections

Female C57BL6 8-12 weeks (young) and 14-20 months (old) were purchased from Charles River (C57BL/6NCrl strain). 1x10^5^ murine melanoma cells (5555, Yumm1.7) in 50μl PBS were injected in left bottom mammary fat pad of the mice. All cell lines were tested for mycoplasma and mouse hepatitis virus (MHV) before injection. The study was performed over two experimental cohorts. Tumor volume was measured over a period of 60 days. First cohort, 5555 contained 8 animals per group and Yumm1.7 contained 4 animals per group. Second cohort, both 5555 and Yumm1.7 contained 3 animals per group. Tumor volume and the health of the mice was observed every two days, followed by once per day measurements once the tumor volume reached 700mm^3^, and tumor volume did not exceed 1500mm^3^, the guideline set by the Committee of the National Cancer Research Institute as stipulated by AWERB. Animals were culled by schedule 1 when tumors reached 1500mm^3^ limit or the tumor interfered with the quality of life. Full body autopsy including brain, liver, heart, lungs, liver, spleen, kidney, and suprarenal (where possible) was conducted on all our mice. The primary tumor from the mammary fat pad was collected either half was fixed in 10% formalin, a quarter snap frozen and stored at -150°C and a quarter stored in PBS at 4°C for dissociation. The lungs, heart, liver, lymph nodes, spleen, kidney, and skin of each mouse was collected and fixed at 10% formalin. One kidney and the skin were snap frozen and stored at -150°C for future use. Fixed tissue samples embedded in paraffin and stained with H&E. No animals were excluded from the analysis.

#### Intracardiac injection

For intracardiac injection, 8–12-week-old female NSG mice were purchased from Charles River. Intracardiac injections were carried on cell lines either treated with secretomes *in vitro* or following single cells obtained following dissociation of mammary fat pad injected cells. For A375, MeWo, A2058 and lipid stripped cells, cell lines were treated with serum free media (control), young and old adipocyte secretome, and synthetic PC, or Ceramide, Cleanascite *in vitro* prior to injection. 5x10^4^ of human melanoma cells or dissociated mammary fat pad tumor cells were resuspended in 100ul of PBS. Cells were injected using the blind approach. The chest cavity of the mice is measured, midpoint identified, and the injection made at the left at 2mm around the left ventricle of the heart. Body weight loss and health of the mice were monitored closely. To avoid sudden death of animals, sudden and continuous weight loss with ill health was used to determine the end point of the study. Animals were culled by schedule 1 when the tumors interfered with the quality of life. All animals with intracardiac injections within an experiment were culled at the same time, and only exceptionally within 72h. Full body autopsy including brain, liver, heart, lungs, liver, spleen, kidney, and suprarenal (where possible) was conducted on all our mice. Collected organs were fixed at 10% formalin. One kidney and a skin sample were snap frozen and stored at -150°C for future use. Fixed tissue samples were embedded in paraffin and stained with H&E and for A2058 cells with anti GFP. Two animals were excluded from the analysis due to incorrect delivery of melanoma cells exclusively to the heart walls instead of the heart chambers.

#### Tail vein injection

For tail vein injection, 8–12-week-old female NSG mice were purchased from Charles River. Single cells dissociated from tumors grown in either aged or young mammary fat pad were used for the study. 5x10^4^ cells were resuspended in 100ul of PBS and injected into the tail vein of the mice. Body weight loss and health of the mice were monitored closely. To avoid sudden death of animals, sudden and continuous weight loss with ill health was used to determine the end point of the study. Animals were culled by schedule 1 when the tumors interfered with the quality of life. Full body autopsy including brain, liver, heart, lungs, liver, spleen, kidney, and suprarenal (where possible) was conducted on all our mice. Collected organs were fixed at 10% formalin. One kidney and a skin sample were snap frozen and stored at -150°C for future use.

#### Administration of drugs to animals

ONC201, NAC, IL6Ri are administered to the mice following the injection of cancer cells in our study. Both ONC201 and NAC was administered in drinking water on the day of injection and IL6Ri- 100μg (BioX cell, BE0047) was administered intraperitoneally every 3 days. IgG2a control for IL6Ri was not used as the study was conducted in NSG mice.

Stock NAC was diluted in 250mL drinking water for treatment concentration of 200mg/kg/day. ONC201 (ApexBio, A8724) was diluted in DMSO at 50mg/ml of ONC201 was diluted in 1000mL drinking water to an administration concentration of 10mg/kg/day. All drinking water was made fresh and changed every two days. Drinking volume was monitored to assure the volume of water consumed was not altered by any treatments.

#### Collection of secretomes and melanoma cell exposure

Differentiated adipocytes were washed twice with PBS and cultured in 2ml serum free DMEM media for 48 hours. The secretomes were collected and centrifuged at 3000 rpm for 5 minutes, aliquoted and stored at -80°C until further use. Secretomes were collected in DMEM media and returned to adipocytes’ maintenance media. To avoid stressing the adipocytes, secretomes were only collected once/ week and rested for at least three days before proceeding with another collection. For IL-6 measurement in secretome, melanoma cells were seeded at 70-80% confluence overnight and treated with respective synthetic compounds diluted in serum free media for 24hours. Following incubation, supernatant was carefully collected in a falcon tube and centrifuged at 3000rpm for 5 minutes to get rid of any debris. The supernatant was then used for ELISA.

#### Lipid strip treatment

For experiments requiring secretomes stripped of lipids, lipids were removed using Cleanascite Lipid Removal Reagent (Biotech Support Group, X2555-100) on the secretomes. Cleanascite was mixed well at room temperature and incubated with secretomes at 1:5 ratio for 10 minutes with gentle shaking. Samples were centrifuged at 1000x g for 15 minutes and the supernatant collected. Lipid stripped control media was fresh prepared in a similar manner prior to each experiment. Melanoma cell lines were seeded at 70-80% confluence a day prior to the treatment. Prior to treatment, cells were washed twice with PBS and treated with adipocyte secretomes. Cells were treated with adipocyte secretomes for 24-48 hours and serum free DMEM was used as a control.

#### Intracellular lipid staining

Fluorescent visualization of intracellular lipids was performed using BODIPY 493/503 (Cayman Chemical, 25892). BODIPY was dissolved in DMSO to 5mg/ml. Cells were incubated with 1μg/ml BODIPY in serum free media and incubated at 37°C for 30 minutes. Cells were washed with PBS twice before imaging. Cellular uptake of PC was monitored using Cy5 labelled PC (Avanti Lipids, 850483). Cells were treated with Cy5-PC at 1μM with 20μM Hoechst 33342 (Thermo Fisher, 62249) and 10μM CellTracker Orange CMRA (Invitrogen, C34551) in serum free media and incubated in 37°C for 30 minutes. Cells were washed with PBS twice before imaging. All imaging was performed using an Opera Phenix (Perkin Elmer) with Zeiss C-Apochromat X63 water immersion objective NA1.15 WD 0.6mm, with at least 20 fields of view per well. Images were processed in Harmony software (Perkin Elmer, Version 6.9).

#### Intracellular mitochondrial staining

For the detection of intracellular active or total mitochondria, melanoma cell lines wells were seeded in 96 well plates and treated with the secretomes, or lipids, for 24 hours. After treatment, cells were incubated with 200nM Mitoview 633 (Biotium, BT70055) for detection of active mitochondria, 20μM Hoechst 33342 (Thermo Fisher, 62249), and 10μM CellTracker Orange CMRA (Invitrogen, C34551) in serum free media and incubated in 37°C for 30 minutes. For total mitochondria, cells were incubated with 200nM MitoTracker Green Dyes (Invitrogen, M7514), 20mM Hoechst 33342 (Thermo Fisher, 62249), and 10mM CellTracker Orange CMRA (Invitrogen, C34551) in serum free media and incubated in 37°C for 30 minutes. After incubation, cells were washed with PBS and serum free media replaced. Cells were imaged using an Opera Phenix (Perkin Elmer) with Zeiss C-Apochromat X63 water immersion objective NA1.15 WD 0.6mm. Images were processed in Harmony 6.9 and average mitochondria intensity per well were used to measure the signals.

#### Metabolism assays

Metabolic functions were assessed in cell using the Seahorse XFe96 Analyser system (Agilent). Cells are treated with all the conditioned media 24 hours before the experiment. On the day of experiment, cells were washed twice with PBS and incubated in Seahorse XF base medium (Agilent, 103335-100) supplemented with 2 mM L-glutamine, 10 mM glucose, and 1 mM sodium pyruvate, and incubated in a non-CO2 incubator at 37°C for 1 hour. Oxidative phosphorylation and mitochondrial function were assessed in cell lines using the Seahorse XF Cell Mito Stress Test kit (Agilent, 103015-100). Glycolysis was measured using the Seahorse XF Cell Energy Phenotype Test kit (Agilent, 103325-100). For all assays, to normalize for cell number, after the assay was complete media was aspirated from the wells and cells lysed in 40μl NP-40 on ice for 30 minutes. NP-40 was prepared by mixing 150nM sodium chloride, 1% Triton X-100 and 50mM Tris pH 8.0. 10μl of the protein lysate from each well was quantified using the Pierce BCA Protein Assay Kit Absorbance was measured using SpectraMax M5 plate reader (Molecular Devices) at 562nm. Wave software (Agilent, version 2.6.1.53) was used to process the data and generate results.

#### Immunofluorescence co-localisation imaging

Melanoma cells were grown on a circular coverslip (Menzel–GlASER) in a 6 well plate dish and treated with secretomes a day prior to fixation. Cells were fixed with 4% PFA containing 4% of sucrose for 15 min followed by permeabilization with 4% PFA with 4% of sucrose and 0.1% triton for 5 min. Cells were then incubated with pAKT-Ser473 (1:500, Cell Signalling, 4060) and Tomm20 (1:1000, abcam, ab56783) primary antibodies for overnight at 4°C. Cells were then washed with 1XTBST for an hour and incubated with secondary antibody (Goat anti-Mouse Alexa Fluor 488, 1:2000, Thermo Fisher, A32723, Goat anti-Rabbit Alexa Fluor 555, Thermo Fisher, A32732) for 2 hours and washed again with 1X TBST for 2 hours. Cells were then placed on glass slide (J.Melvin freed Brand, 7625M) using 20ul of mounting medium (Vectashield, H-1000) and dried at 37°C overnight covered in aluminum foil. Samples were imaged with conventional confocal mode on a Zeiss LSM880 inverted microscope equipped with Plan-Apochromat 63x NA 1.4 Oil objective lens (Zeiss), controlled by Zeiss Black software (version 2.3). Multi-channel Images were acquired with sequential track mode. DAPI was excited with 405nm line diode laser (Coherent) and collected with PMT detector from spectrum range 410 – 480nm; Alexa Fluor 488 was excited with the 488nm line Argon laser (Lasos) and collected with GaAsP detector from spectrum range 500 – 550nm. Alexa Fluor 555 was excited with 561nm line DPSS laser (Lasos) and collected with PMT detector from spectrum range 570-650nm. Z-series optical sections were collected with a step-size of 0.323 micron driven by Z Piezo WSB 500 stage (Zeiss).

#### Mitochondria extraction

Mitochondrial extraction was conducted by adapting Frezza et al. (2007)^31^ protocol on isolation of functional mitochondrial (Frezza, et al., 2007). Upon treatment with secretomes, melanoma cells were washed with PBS twice and trypsinised, collected and washed twice with PBS. Cells were transferred to a 50ml centrifuge tube and centrifuged at 600x g at 4°C for 10 minutes. Cells were resuspended in 3ml ice cold IBc buffer (10mM Tris-MOPS, 1mM EGTA-Tris, 200mM Sucrose, pH 7.4). 2ml syringe plunger was used as pestle in 50ml falcon tube and vortexed at 1000rpm for 5 minutes. Falcon tube was occasionally put in ice to prevent protein damage. The plunger was removed, and cells centrifuged at 600x g at 4C for 10 minutes. The supernatant was collected and centrifuged at 7000x g for 10 minutes at 4°C. Pellets were washed with 200ul Ibc buffer and centrifuge again at 7000x g for 10 minutes at 4C. Supernatant was discarded, and mitochondrial pellet resuspended in 40μl of the remaining buffer. Mitochondrial protein concentration was calculated using 1μl of solution in Pierce BCA Protein Assay Kit (Thermo Fisher, Cat no:23225). Samples were diluted in Laemmli Buffer (Bio-Rad, 1610747) with Beta-mercaptoethanol, denatured at 95°C for 5 minutes.

#### Western Blot

Protein was extracted from cells with NP-40 lysis buffer supplemented with 1X phosphatase inhibitor (Roche, 4906845001) and 1X protease inhibitor (Roche, 11836153001) on ice for 30 minutes and then centrifuged at 13000 rpm for 15 minutes at 4°C. The supernatant containing protein was collected and stored at -80°C until further use. Protein concentration was quantified with Pierce BCA Protein Assay Kit (Thermo Fisher, 23225). 40-100ug of protein was diluted in Laemmli Buffer (Bio-Rad, 1610747) with Beta-mercaptoethanol, (Sigma, M6250-M6250) denatured at 95°C for 5 minutes, loaded onto Mini-PROTEAN TGX gels (Bio-Rad, 4568084) with Precision Plus Ladder (Bio-Rad, 1610374) and ran in 1X TGS buffer at 100V (Bio-Rad, 1610732). Samples were transferred to nitrocellulose membranes using the TransBlot Tubro system (Bio-Rad, 170-4270) and protein visualized with Ponceau Stain (G-Biosciences). Membranes were washed in 1X Tris Buffer Saline with 0.1% Tween 20 (TBST) and blocked in 5% BSA in TBST for an hour and incubated with primary antibodies diluted in 5% BSA TBST over night at 4°C (pAKT-Ser473 1:500, Cell Signaling, 4060, pAKT-Thr308 1:500, Cell Signaling, 4056, AKT 1:1000, Cell Signaling, 9272, pmTORC2 1:1000, Cell Signaling 2972, total MTOR 1:1000, Cell Signaling, 5536, pSTAT3^Tyr705^Cell Signaling 9131 1:1000, STAT3 Cell Signaling 9132, 1:1000), BCL2 1:1000 Cell signaling, 15071, beta-tubulin 1:5000, Abcam, ab6046, Cytochrome C 1:1000, Abcam, ab13575, CPT1a 1:1000, Proteintech, 66039-1). Membranes were washed for 1 hour in 1X TBST at room temperature and incubated with secondary antibodies (Anti-Rabbit IgG, HRP-linked Antibody, Cell Signaling, 7074 or Anti- Mouse IgG, HRP-linked Antibody, Cell Signaling, 7076 in 1:5000 ratio.) at 1:5000 in 5% BSA TBST for 2 hours at RT. After incubation, the membrane was washed again with 1X TBST for an hour and exposed with ECL western blot substrate (Promega, w1001). Quantification was performed using ImageJ 1.52as. Frames of same area were aligned, and intensity graph generated per blot. Area under the graph was calculated and divided by control area to study the relative band strength per group.

#### IHC and SA beta galactosidase staining

Paraffin embedded MFP tissues were cut into sections and stained for different murine proteins: FASN (Cell Signalling Technology, cat no.3180S), CD36 (Cell Signaling Technology, Cat no. 28109s), PPARγ (Cell Signalling Technology, cat no. 2435s), and FABP4 (Abcam, ab, 92501).

SA-beta-Gal activity was assessed in differentiating primary human aged and young adipocytes using the senescence detection kit (Abcam, ab65351). Cells were seeded and differentiated in a 96 well plate. Cells were washed with PBS and fixed with a fixative solution for 10 minutes. Cells were washed with PBS and incubated with staining solution overnight at 37C. Staining was visualized under the microscope at 10X and images captured. Quantification of the staining was carried out by counting positive blue staining per image. All SA-β galactosidase ^positive^ cells in the image were counted within a representative image per cell line.

#### Lentiviral knock down of CPT1a

Knockdown of CPT1a expression in A375 and SKMEL28 was performed using shRNA Lentiviral Particles (Santa Cruz Biotechnology). For CPT1a shRNA knockdown CPT1a shRNA (h) lentiviral particles (sc-40376, shRNA) were used alongside a scramble control, control shRNA lentiviral particles A (sc-108080) A total of 5 x10^4^ cells were cultured media with 5ug/ml polybrene (Santa Cruz Biotechnology, sc-134220). Lentiviral particles were added to cells and incubated overnight. Media containing lentiviral particles and polybrene were removed and incubated in DMEM overnight before performing selection of transfected cells using increasing concentration of puromycin (Sigma Aldrich, P8833-10MG) over 72 hours. Once cells were stably grown, cells were cultured in regular DMEM media. Control cells transfected with shRNA lentiviral particles A were also cultured without puromycin to use as control to validate the knockdown in western blot.

#### Cell treatments

PC (Egg-PC, Avanti Polar Lipids, 840051) was resuspended in DMSO at 20mM and diluted to 5μM in DMEM media for treatment for 24 hours, with DMSO only as vehicle control. Cy5-PC (Avanti Polar Lipids, 850483C) was purchased at stock concentration of 190mM and was diluted to 1mM final concentration in DMEM. JR-AB2-011 (MedChem Express, HY-122022) was prepared in DMSO at 5mM and diluted to 100nM in DMEM or secretomes for metabolism and ROS assays or 500nM for proliferation assays. AKT inhibitor-LY294002 (Cambridge Bioscience, 70920-5mg-CAY) was prepared at a stock concentration of 5mg/ml in DMSO and then diluted in DMEM or secretomes to 5μg/ml for treatment. BCL2 inhibitor ABT199 (ApeXBio, A8194) was diluted in DMSO to a concentration of 10mM and diluted in DMEM or secretomes to a final concentration of 1μM. Salvianolic acid (Cayman chemical, 9001577) was diluted in DMSO at a stock concentration of 14mM and diluted in DMEM or secretome to a final concentration of 20μM. CPT1a inhibitor, Etomoxir (MedChemExpress, HY-50202) was purchased and diluted in DMSO to a final stock concentration of 5mM and diluted to 10μM in secretome or DMEM. Ceramide (ThermoFisher, D-7540) was purchased at a stock concentration of 354mM and was diluted to a final concentration of 100nM in DMEM. All experiments contained vehicle controls. Biological replicates reflect the different batches (different types, differentiated at different dates) of adipocytes.

#### Lipidomics

Lipidomics Mass spectrometry was performed for lipid analysis using the Folch method. Briefly, 1ml of adipocyte secretomes were transferred to Eppendorf tubes with 5mm stainless steel beads (Qiagen, 69989). 400 μl of chloroform and methanol solution (2:1 ratio) was added to the secretome and vortexed. The samples were placed in a Qiagen TissueLyser II for 30s at 20Hz. 150ul of stable isotope labelled LIPID internal standard was added and vortexed. 600ul of the chloroform:methanol solution was added to the mixture and vortexed. 400μl of HPLC water was added, vortexed ad then centrifuged at 21000x g for 5 minutes to separate the aqueous and organic layers. The organic layers were removed and the aqueous layer resuspended in 1ml chloroform:methanol solution, vortexed and lysed for 30 seconds at 20HZ. The samples were centrifuged at 21000xg for 5 minutes. The organic layer was then injected into the mass spectrometry for analysis by the collaborator. Lipidomics mass spectrometry on mammary fat pad tissues were conducted by Dr Jair Marques Junior. Mammary fat pads were harvested from young and aged C57BL/6 mice and sent for analysis to University of Edinburgh. Relative lipid quantification was measured between young and aged mammary fat pads.

#### Proteomics extraction

Each 2ml media sample was split equally into 10x 200ul aliquots. 800ul of ice cold acetone was added to each aliquot followed by brief vortex and incubation at -20 degrees C for 30 mins. Samples were centrifuged at 13,100g at 4°C for 20 mins before the supernatant was gently aspirated away and discarded. The precipitated pellets were resuspended in 20ul 5 % SDS, 50mM TEAB followed by water bath sonication for 10 mins. Proteins were reduced by addition of TCEP to a final concentration of 5mM and heated at 55°C for 15 mins before cooling to room temperature. Proteins were alkylated by addition of MMTS to a final concentration of 15mM and incubated at room temperature for 10 mins. Proteins were precipitated by addition of phosphoric acid to a final concentration of 1.2% followed by briefly vortexing. Each aliquot was then then processed using STRAP mini units according to the manufacturer’s instructions (https://protifi.com/pages/s-trap). Proteins were digested by the addition of 5ug of sequencing grade trypsin (Sigma) in 125ul 50mM TEAB with incubation at 37°C for 18 hours. STRAP eluted peptides from each aliquot constituting a single sample were then pooled and dried in a vacuum centrifuge

#### Tumor dissociation protocol

For dissociation of tumors into single cells, approximately 30mg of tumor was chopped into fine pieces using a scalpel and washed twice with PBS. Chopped tumors were transferred into a 50ml Falcon tube containing serum free DMEM media with collagenase IV (100U.ml, Worthington Biochemical) and DNase I (0.1mg/ml). The mixture was incubated in 37C water bath and vortexed every 10 minutes. Following 30 minutes incubation, serum free media was topped up to 50ml. The tube was then centrifuged at 600rpm for 5 minutes and the supernatant discarded until only a white pellet was visible. The pellet was resuspended in either PBS for injection or media for downstream processing. Cell viability was calculated using trypan blue. Cell viability was improved by using the dead cell removal kit (Miltenyi Biotech,130-090-101).

#### Proliferation assay

Proliferation of melanoma cells was quantified using the confluence assay on an IncuCyte S3 (Essen Bioscience). Cells were plated in clear bottom 96-well plates in triplicate wells and treated with adipocyte secretomes, synthetic PC, Lipid stripped secretomes. Plates were incubated at 37°C and imaged in real time for phase-contrast images over 48-72 hours at 2-hour intervals with 4 images per well, which allowed controlling for cell size. Phase masking and calculation of confluence used to compare growth rate of cell lines in each condition using the IncuCyte Software (Essen Bioscience, 2020C Rev1). Relative confluence was calculated by dividing the confluence of the cells with the initial confluence of the cells.

#### Intracellular ROS assays

Melanoma cell lines were seeded in a 96 well plate in triplicate (Perkin Elmer CellCarrier Ultra, 6055300) overnight prior to treatment with secretomes, synthetic lipids or inhibitors. Intracellular ROS was detected with 12.5μM CellROX Green (Thermo Fisher, C10444) and mitochondrial ROS was detected with 5μM MitoSOX Red mitochondrial superoxide indicator (Thermo Fisher, M36008). Plates were incubated at 37°C and imaged in real time for phase-contrast, and red (MitoSOX) and green (CellROX) fluorescent channels over 24 hours at 0.5 or 1-2-hour intervals in an IncuCyte S3. Analysis was conducted using IncuCyte Software (Essen Bioscience, 2020C Rev1). Four images per well was used to measure the Total integrated intensity (RCU x μm^2^/Image) to measure the signals.

#### GSH/GSSG-Glo ratio assay

GSH/GSSG ratio of cells treated with adipocyte secretomes, or synthetic lipids was carried out using Promega GSG/GSSG-Glo Assay Kit (Promega, V6611). 10,000 A375 cells and 5000 SKMEL28 and MeWo were seeded in a 96 well plate (Corning, 3915) a day prior to the experiment. Cells were treated with either secretome or synthetic lipid for 6 hours at 37°C. Media was aspirated from the well following treatment and cells lysed using the GSH/GSSG lysis kit. Cells were treated as per manufacturer’s instructions and the luminescence measured using Spectra Max M5 plate reader (Molecular Devices). All the conditions were performed in duplicate.

#### Drug administration *in vitro*

For this study, ONC201 was diluted to 1mM stock solution in DMSO. Cells for ONC201 treatment was seeded a day before the experiment. The cells were washed with serum free media twice. ONC201 was diluted to desired concentration in serum free media. Cells were then treated with ONC201 either overnight for mitochondrial activity or proliferation assessed over time in incucyte for 48 hours. BCL2 inhibitor – ABT199 was purchased from MedChem Express (HY-15531) and diluted in DMSO. mtorc2 inhibitor -JR-AB2-011 was purchased from MedChem Express (HY-122022) in DMSO solution. PI3K inhibitor -LY294002 was also purchased from MedChem Express (HY-10108) and diluted to a stock solution of 5mg/ml in DMSO.The drug was diluted in serum free media or conditioned secretome prior to administration to cancer cells.

#### Phosphatidylcholine content analysis

PC content in tumor and cells were analyzed using a commercial Phosphatidylcholine Assay Kit (Abcam, ab83377). For intracellular PC content analysis, post incubation with either secretome or synthetic lipids administration, cells were harvested and washed twice in cold PBS. Cells were then resuspended in assay buffer and homogenized on ice for 10 minutes. The samples were centrifuged at top speed at 4°C for 5 minutes and supernatants used for analysis.

All the tumors were cut into small pieces, one for dissociation, one for snap freezing and another for histology analysis. Snap frozen tumor samples were used for qPCR and PC content analysis. Tumors were thawed on ice. Tumors were dried with a tissue to remove any excess water from ice. For PC content analysis, tumor size was weighed and recorded before processing the samples. The samples were then digested as per the kit’s instructions. The calculated values were normalized to the weight of the samples.

#### Plasma Isolation

For isolation of plasma, 70-100μl of blood was tail veined three days following intracardiac injection of Aged- MFP melanoma cells using EDTA tubes. Blood was centrifuged at 400xg at 4°C for 10 minutes to isolate supernatant plasma. Plasma was carefully transferred into eppendorff tubes and stored at -80°C until further use.

#### ELISA IL-6 measurement

Murine and human IL6 levels in blood and culture media were measured using the manufacturer’s instructions (R&D systems, SM6000B and DY206 respectively). Briefly, for the detection of murine IL6 in the blood of a mouse, blood was centrifuged for 1200rpm for 10 minutes at 4°C. The supernatant plasma was carefully separated from the blood and stored at -80°C. For the detection of human Il6 in culture media of human melanoma cells upon the exposure to synthetic lipids and secretomes, cells were incubated in respective media for 24 hours and supernatant collected, centrifuged at 3000rpm and stored at -80°C. Neither plasma nor culture media was diluted prior to the experiment.

#### RNA extraction, cDNA synthesis and qPCR analysis

For qPCR analysis, RNA was extracted using Trizol solution (Invitrogen,12044977) Cells were homogenized with 1ml of Trizol reagent and incubated at room temperature (RT) for 5 minutes. Phase separation was performed with 200μl of chloroform and samples were mixed vigorously and incubated at RT for 5 minutes. The mixture was centrifuged at 12000x g at 4°C for 15 minutes and the aqueous phase transferred to a new tube. To precipitate RNA, 500μl of isopropanol was added and mixed and incubated at RT for 20 minutes. The solution was centrifuged at 12000x g at 4°C for 15 minutes. The RNA pellet was then washed with 1 ml of 70% ethanol centrifuged at 7000x g for 5 minutes. The pellet was air-dried at RT and dissolved using RNAse free water (Thermo fisher, AM9938). The quantity and quality of RNA were assessed using BiodropmLITE (Advanced TNI Company Limited). cDNA was synthesized from total RNA extracted using Qiagen Omniscript reverse transcriptase kit (Qiagen, 205113). 1μg of RNA was used for cDNA synthesis with 5mM oligo dT, 1mM dNTPs, 2x buffer with a final volume of 20μl with nuclease-free water and incubated at 37°C for one hour. cDNA was then diluted in 1:10 in nuclease-free water. For qPCR reactions were performed with 1x SYBR Green (Sigma Aldrich, 4913850001), 0.5μM forward and reverse qPCR human primers and murine primers (Table S7) and 2μl of 1:10 cDNA with a total volume of 10μl with nuclease-free water per well in optical 96-well plates (Applied Biosystems, N8010560). All qPCR reactions were performed in triplicate, and the amount of target gene expression was calculated by the relative quantification method with respective housekeeping gene as the normalization.

### Quantification And Statistical Analysis

#### Human adipocyte analysis

To score adipocyte heterogeneity in human samples, we analyzed the variability in adipocyte diameter in a cohort of consecutive skin biopsies of patients with epidermal, dermal disease, or tumor-adjacent normal skin, biopsied at the department of pathology, Salford Royal NHS Hospital Pathology (L.M., A.V.), UK. Patients with inflammatory processes in the dermis and hypodermis, hypodermal tumors, scars, crush artefacts and lipidic necrosis were excluded. Areas immediately adjacent to tumors were excluded. Body mass index was available for a subset of cases. L.M. was blinded for the hypothesis. We ranked the variability in diameter between adjacent adipocytes in the subcutis in x20 high power fields (HPF). We scored heterogeneity as low (1) when adipocytes presented long diameters within 20% length of each other, medium (2) when diameters varied 20-50%, and high (3) when some adipocytes presented vastly different sizes, >50% variability in length, and determined year of birth. IRAS 216310, REC 16/LO/2098. To score adipocyte size, E.N. and E.M. measured the long and short diameters of 6 adipocytes randomly selected with the aid of a grid from normal wide local excisions of young (n=10) and aged (n=10) patients matched for anatomic site (abdomen) and normal body mass index, from consecutive specimens seen at Instituto Valenciano de Oncología, Valencia, Spain. Average size per adipocyte was calculated as long x short diameter, and averages of six adipocyte sizes calculated per patient. To score adipocyte count, all adipocytes that were entirely visible within randomly selected n=4 x20 HPF per patient were counted. The measurements were undertaken by dermatologists blinded for the hypothesis (E.M., E.N.). All patients actively consented for research under local and national ethics.

#### Histology analysis

Mouse autopsy collection of lungs, liver, kidney, spleen, renal and adrenal tissue was performed for *in vivo* experiments. Tumor burden was scored as a percentage replacement of the normal organ parenchyma: and scored as 0: absent tumor, 1: 1-5%, 2: 6-10%, 3: 11-25%, 4: 26-50%, 5: 51-75% and 6: >75% tumor replacement of the parenchyma. Tumor burden in the graph is the sum of all tumor burden scored in each organ. Metastasis distribution between lungs and liver was calculated by dividing tumor burden in the organ by the total tumor burden. A subset of samples were scored by S.G, L.M. and A.V, and 39 samples were scored to assess interobserver consensus by L.M. and A.V., which showed high interobserver agreement (L.M./A.V kappa interobserver agreement linear weighted kappa =0.84, where L.M. was blinded for the hypothesis), the remainder were jointly scored by A.V and S.G. Melanoma tropism was scored as the percentage distribution in the lung compared to the liver of the total tumor burden. Two animals in total were excluded, both due to erroneous intracardiac injection delivered exclusively into the heart walls, leading to rapid heart failure, with no metastatic spread of disease in organ parenchyma.

#### Metastasis HALO analysis

Whole slide imaging was performed using the Olympus VS200 MTL (Olympus Tokyo, Japan), in conjunction with an Olympus UPLXAPO20X (NA 0.6): 0.274 μm/pixel objective lens.

Focus points were chosen to permit a well-defined image across the whole tissue, using intervention to move focus points to any problematic areas. The data was captured on multiple axial planes, and then combined using a depth of focus algorithm to maintain focus and render a three-dimensional image into two dimensions. Image capture was via a Orca-Flash4 (Hamamatsu Photonics, Germany) where each field of view was captured at 2048x2048 pixels, all under control from Olympus ASW software. Data was saved directly in the Olympus.VSI format thus maintaining image capture metadata and stored on a read only server.

To validate our blinded histological approach to tumor scores, we selected randomly 2 *in vivo* experiments. For these, we took images, which were analyzed by Halo 4.0.5107.407 (Indica Labs, New Mexico USA), with Random Forest machine learning classifiers applied to identify normal and tumor areas. Metastasis burden is calculated by dividing tumor area to total lung or liver area (Figure S7I). Metastasis burden is calculated by comparing the respective percentage of lung and liver burden. Experiments show overlapping results with Figure 4.

#### Proteomics quantification

The LCMS data were acquired using an RSLCn HPLC coupled to an Orbitrap Lumos. The separation was performed on Pepmap Easyspray C18 column 25cm 75um ID column using direct injection at a flow rate of 180nl/min. Mobile phase A was 0.1% formic acid and mobile phase B was 80% (v/v) acetonitrile0.1% formic acid. A gradient of 4%-40% mobile phase B over 45 mins was used to separate peptides before they were sprayed into the EasySpray source and a voltage of 1.8KV. The Mass Spectrometer was operated in data dependent mode. Briefly, MS1 was collected in the Orbitrap detector between m/z of 400-1200 at a nominal resolution of 120K (at m’z 200). All multiply charged ions were targeted for MS2 using HCD with a normalized collision energy of 30% orbitrap resolution of 30,000 resolution and a maximum fill time of 54ms. Each sample was injected 5 times with identical MS1 parameters but in each of the 5 injections, MS2 m/z was limited to 20% of the available populated mass range to increase qualitative penetrance whilst maintaining quantitative information in each technical replicate.

#### RNA sequencing

For RNA sequencing, 2x10^5^ MeWo cells were grown in a 6 well plate and 2x10^6^ A375 cells were grown in a T75 well plate. Cells were washed twice with PBS and treated with either DMEM (control), old adipocyte secretome (OA) and young adipocyte secretome (YA) respectively. Cells were incubated for 24 hours and trypsinised for RNA extraction kit. RNA from cells were extracted using Qiagen RNA extraction kit (Qiagen, Cat no. 74104) as per manual instructions. As for tissues, tissues were chopped into small pieces and digested in buffer using tungsten carbide beads (Cat no. 69997, Qiagen) and tissue lyser for 30s. Lysed tissues were immediately processed as per manual’s instructions (Cat no. 74014, Qiagen). 1μg of purified RNA was submitted to either or the core facilities to run RNA sequencing analysis. A375 and murine mammary fat pad RNA sequencing was analysed using DESEq2.For MeWo, GFold (v1.1.4) was used to carry out for differential analysis. Differential analysis on RNA sequencing were carried out to identify differential expressed genes in between groups. Genes upregulated by 1.2 were used to assess pathway analysis in DAVID enrichment analysis.

#### Pathway enrichment analysis

Single cell RNA sequencing (Pozniak et al., 2024)^43^ data was downloaded from KU Leuven Research Data Repository. Single cell data was analysed using Seurat package (version 4.0.2). The data was filtered to only include the subcutaneous metastasis samples before immunotherapy and grouped into young and old based on patients’ age (old>55; young<55). Signature scores for the different pathways were calculated by the *AddModuleScore* function in Seurat. Pathways used:

PC Catabolism = GOBP_PHOSPHATIDYLCHOLINE_CATABOLIC_PROCESS

Phosphatidylinositol Signalling = KEGG_PHOSPHATIDYLINOSITOL_SIGNALING_SYSTEM

S1P Receptor Signalling = GOBP_SPHINGOSINE_1_PHOSPHATE_RECEPTOR_SIGNALING_PATHWAY

Glycolysis = REACTOME_GLYCOLYSIS

#### Statistical analyses

Data collection was performed with Microsoft Excel (64-bit, Microsoft). For *in vitro* studies statistical analysis was performed in GraphPad Prism (version 8.2.0, GraphPad Software, Inc.). For comparisons between two groups Mann Whitney tests were Anova was used as mentioned in the legends. A p-value <0.05 was considered significant.

## Resource Availability

### Lead contact

Requests for information and resources should be directed to Amaya Viró s (amaya.viros@cruk.manchester.ac.uk).

### Materials availability

This study did not generate new reagents.

## Supplementary Material

Supplemental information can be found online at https://doi.org/10.1016/j.ccell.2025.04.001.

Supplemental data

## Data Availability

RNA-seq data were deposited at GEO under accession numbers GSE292627, GSE292628, and GSE292629 and are publicly available.

## In brief

Gurung et al. show that young subcutaneous adipocytes provide lipids and phosphatidylcholine to melanoma cells, which activates PI3K-AKT, OXPHOS, and oxidative stress. High OXPHOS reduces metastatic burden and associates with lung tropism. Conversely, aged subcutaneous adipocytes provide ceramides to melanoma cells, which activates S1P-STAT3-IL-6 signaling, increasing total metastatic burden and liver metastasis.

## Highlights

- Age-dependent adipocyte lipids increase melanoma OXPHOS, which impacts metastasis
- Lipid-rich young skin imposes high OXPHOS and reduces metastasis
- Lipid-scarce aged skin drives lower OXPHOS and promotes metastasis
- Young lipids/phosphatidylcholine drive lung tropism; aged ceramides drive liver tropism
