# Supplementary material for: Stromal lipid species dictate melanoma metastasis and tropism
Source: Cancer Cell. Author manuscript; Available in PMC 2026 Jan 19. (PMC7618647; doi:10.1016/j.ccell.2025.04.001)
Supplement: Supplemental data [file EMS212008-supplement-Supplemental_data.pdf]

Supplemental Information

Stromal lipid species dictate melanoma metastasis and tropism

Shilpa Gurung[1], Timothy Budden[1], Karthik Mallela[1], Benjamin Jenkins[2,3], Alex von Kriegsheim[4], Esperanza Manrique[5,6], David Millán-Esteban[5,6], Isabel Romero-Camarero[7], Fabio Amaral[7], Sarah Craig[1], Pedro Durao[1], Joanna Pozniak[8,9], Laura Stennett[10,11], Duncan Smith[12], Garry Ashton[13], Alex Baker[14], Kang Zeng[14], Gilbert Fruhwirth[10,11], Victoria Sanz-Moreno[15,16], Jair Marques[4], Albert Koulman[2,3], Jean-Christophe Marine[8,9], Tim CP Somervaille[7], Luisa Motta[17], Caroline Gaudy-Marqueste[18], Eduardo Nagore[5,6], Amaya Virós[1,19, 20*]

Content:
Table S1,
Figures S1-S8
Table S7

Table S1: Patient adipocyte donor age, sex, site, source, related to Figure 1

Batch	Age (y)	Sex	Site	Source
P1	43	Female	Chest	Purchased- Lonza
P2	30	Female	Chest	Purchased- Lonza
P3	42	Female	Chest	Purchased- Lonza
P4	37	Female	Chest	Purchased- Lonza
P5	18	Male	L lower leg	Biobank
P6	60	Male	Chest	Purchased- Lonza
P7	91	Male	Scalp	Biobank
P8	79	Male	R anterior thigh	Biobank
P9	69	Male	R buttock	Biobank
P10	76	Male	R Upper Chest	Biobank
P11	41	Female	Chest	Purchased- Lonza
P12	56	Female	Chest	Purchased- Lonza

FIGURE S1

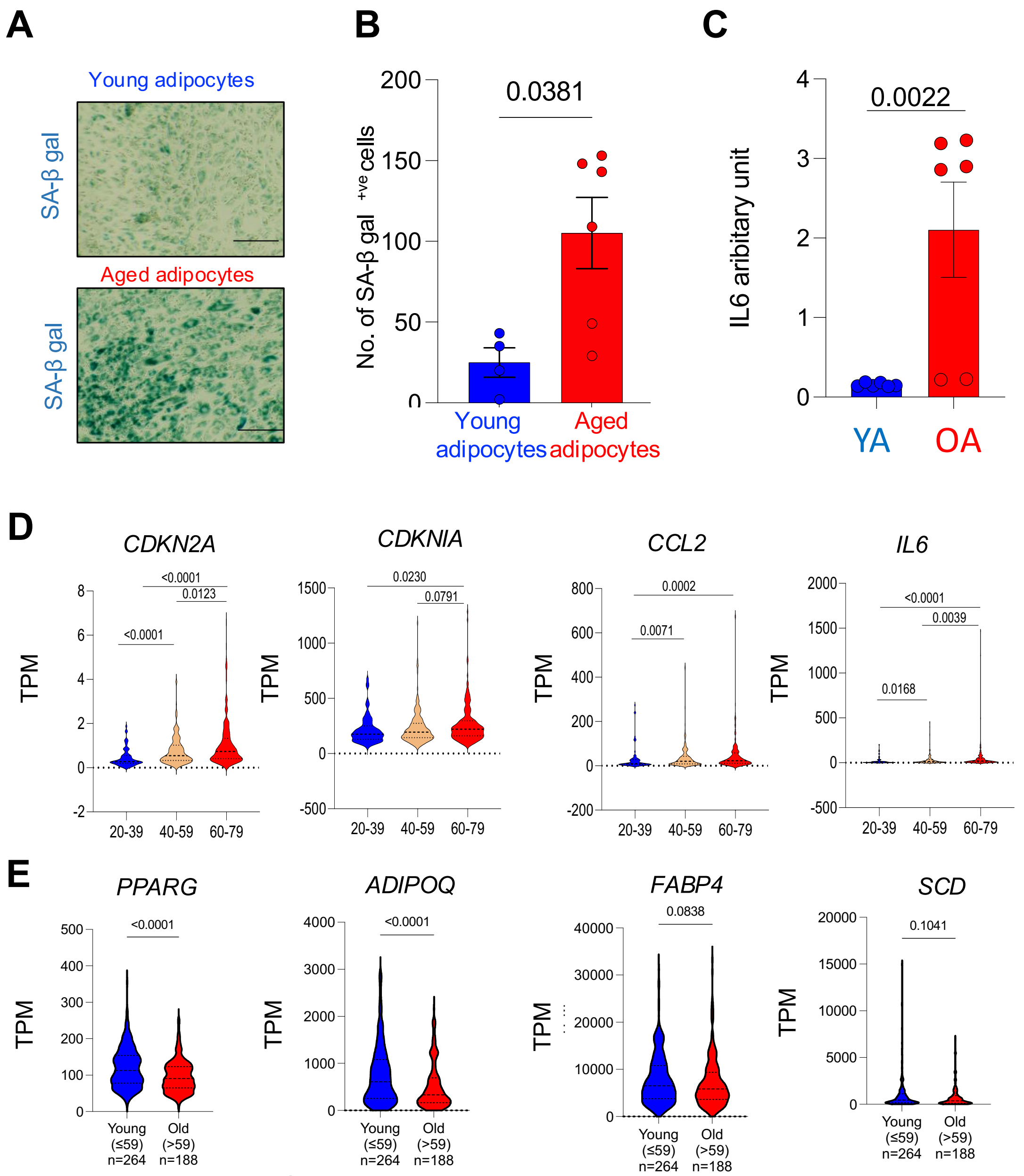

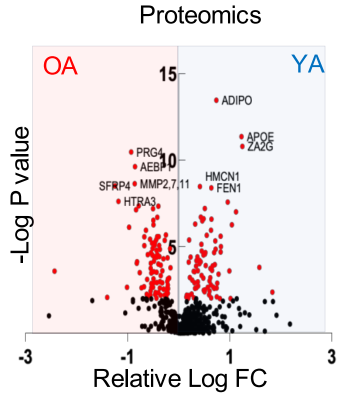

G

Proteins related to Lipid transport and metabolism	Function	Secretion in Young vs Old Secretome (FC)
ADIPO	Regulation of glucose metabolism.	5.46796845
APOE	Binds to lipids to form lipoprotein.	17.0418603
ZA2G	Major histocompatibility complex I molecule and a lipid-mobilizing factor.	17.7001522
PLIN4	Coats intracellular lipid storage droplets	9.1320441
FABP4_HUMAN;MYP2	Fatty acid binding protein 4	4.76327878
FABP5	Fatty acid binding protein 5	2.38

Figure S1: Characterization of Young and Aged human adipocytes (related to Figure 1)
(A) Representative images of SA-β-galactosidase staining of adipocytes. Bar 200 μM.
(B) Graph represents the quantification of the number of SA-β-galactosidase positive cells in young and aged human adipocytes. Data represents mean and standard error, two-sided Mann Whitney U (Young Adipocytes n=4 (blue), Aged Adipocytes n=6 (red)).
(C) IL-6 production in the conditioned media of young (YA) and old (OA) adipocytes. Data represents mean and standard error, two-sided Mann Whitney U (n=6 biological replicates).
(D) Expression (transcripts per million, TPM) of senescent markers (*CDKN2A, CDKN1A, CCL2, Il6*) in young (<40 years, blue), middle aged (40-59, orange), and aged (>60 years, red) subcutaneous adipose tissue from the Genotype Tissue Expression (GTEx) patient cohort. Violin plots include 25th to 75th percentiles and median, ****p<0.0001, two-sided Mann Whitney U.
(E) Expression (transcripts per million, TPM) of adipocyte lineage genes (*PPARG*, *ADIPOQ*, *FABP4*, *SCD*, *FASN*) in young (<60 years, blue) and aged (>59 years, red) in subcutaneous adipose tissue from the Genotype Tissue Expression (GTEx) patient cohort. Violin plots include 25th to 75th percentiles and median, ****p<0.0001, two-sided Mann Whitney U.
(F) Volcano plot representing the differentially secreted proteins detected in young (YA) and old (OA) adipocytes' secretome (n=2). Top 10 proteins are labelled in the graph. Data represents the relative log fold change (FC) and the negative log p value.
(G) Table highlighting the significant lipid proteins present in high amount in young adipocyte secretome.

FIGURE S2

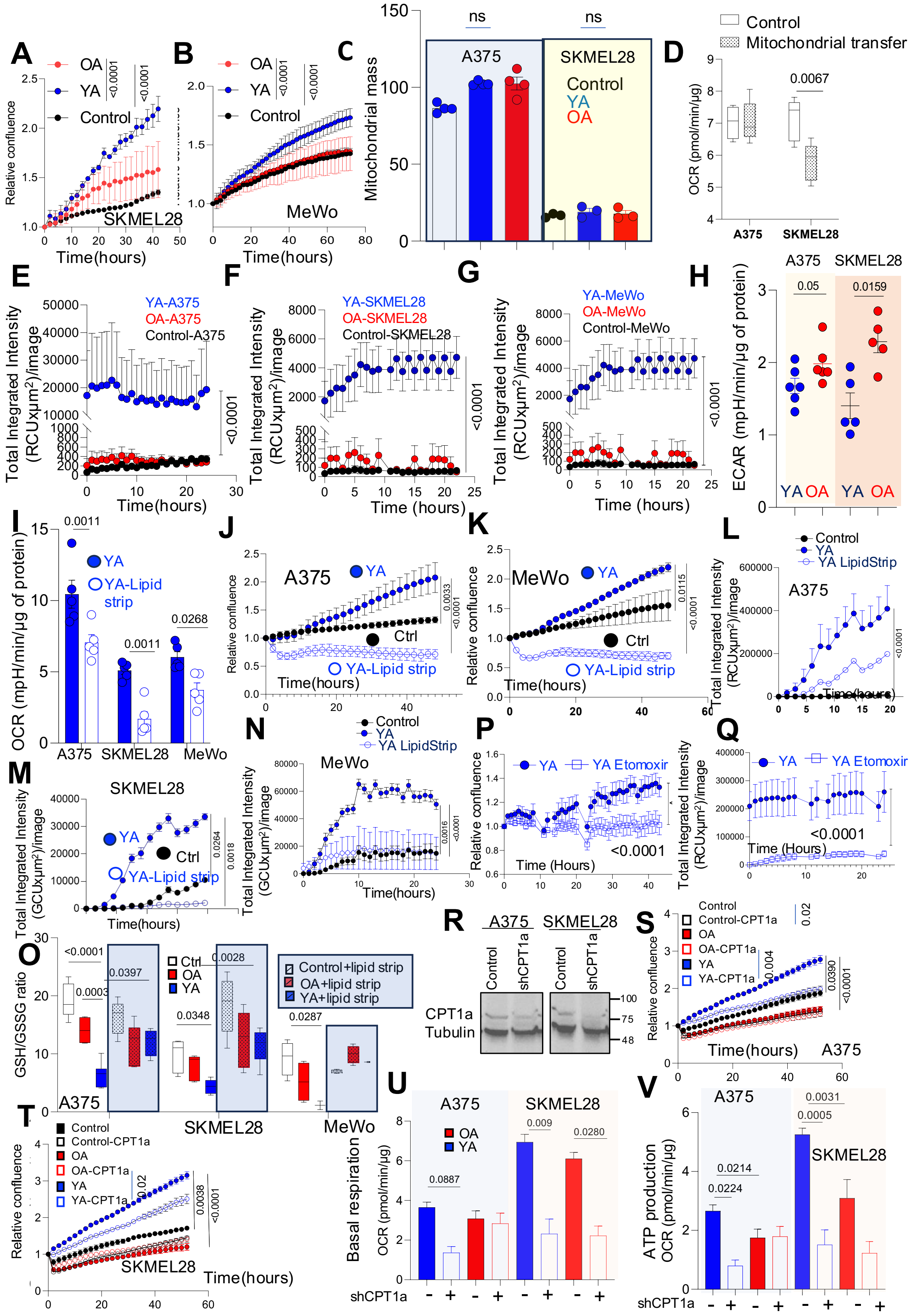
A
OA
YA
Control
<0.0001
<0.0001
Relative confluence
SKMEL28
Time(hours)
B
OA
YA
Control
<0.0001
<0.0001
MeWo
Time(hours)
C
Mitochondrial mass
ns
ns
A375
SKMEL28
Control
YA
OA
D
Control
Mitochondrial transfer
OCR (pmol/min/µg)
0.0067
A375
SKMEL28
E
YA-A375
OA-A375
Control-A375
Total Integrated Intensity (RCUxµm²)/image
<0.0001
Time(hours)
F
YA-SKMEL28
OA-SKMEL28
Control-SKMEL28
Total Integrated Intensity (RCUxµm²)/image
<0.0001
Time (hours)
G
YA-MeWo
OA-MeWo
Control-MeWo
Total Integrated Intensity (RCUxµm²)/image
<0.0001
Time (hours)
H
A375
SKMEL28
ECAR (mpH/min/µg of protein)
0.05
0.0159
YA OA YA OA
I
OCR (mpH/min/µg of protein)
YA
YA-Lipid strip
0.0011
0.0011
0.0268
A375
SKMEL28
MeWo
J
A375
YA
Ctrl
YA-Lipid strip
Relative confluence
Time(hours)
0.0033
<0.0001
K
MeWo
YA
Ctrl
YA-Lipid strip
Relative confluence
Time(hours)
0.0115
<0.0001
L
Control
YA
YA LipidStrip
A375
Total Integrated Intensity (RCUxµm²)/image
Time(hours)
<0.0001
M
SKMEL28
YA
Ctrl
YA-Lipid strip
Total Integrated Intensity (GCUxµm²)/image
Time(hours)
0.0264
0.0018
N
Control
YA
YA LipidStrip
MeWo
Total Integrated Intensity (GCUxµm²)/image
Time(hours)
0.0016
<0.0001
P
YA
YA Etomoxir
Relative confluence
Time (Hours)
<0.0001
Q
YA
YA Etomoxir
Total Integrated Intensity (RCUxµm²)/image
Time (Hours)
<0.0001
O
GSH/GSSG ratio
<0.0001
0.0003
0.0397
Ctrl
OA
YA
0.0028
0.0348
Control+lipid strip
OA+lipid strip
YA+lipid strip
0.0287
A375
SKMEL28
MeWo
R
A375
SKMEL28
Control
shCPT1a
Control
shCPT1a
CPT1a
Tubulin
100
75
48
S
Control
Control-CPT1a
OA
OA-CPT1a
YA
YA-CPT1a
0.02
0.004
0.0390
<0.0001
Relative confluence
Time(hours)
A375
T
Control
Control-CPT1a
OA
OA-CPT1a
YA
YA-CPT1a
0.02
0.0038
<0.0001
Relative confluence
SKMEL28
Time(hours)
U
A375
SKMEL28
OA
YA
0.0887
0.009
0.0280
Basal respiration OCR (pmol/min/µg)
shCPT1a
V
A375
SKMEL28
0.0031
0.0005
0.0214
0.0224
ATP production OCR (pmol/min/µg)
shCPT1a

Figure S2: Adipocytes impose metabolic changes in melanoma cells by age (Related to Figure 2)

(A,B) Proliferation of (A) SKMEL28 and (B) MeWo cells treated with young adipocyte (YA, blue) or old adipocyte (OA, red) secretes or control (black) over 48 hours. Data represents mean and standard error, n=3 biological replicates, two-way ANOVA.**(C)** Total mitochondria (mitochondrial mass) in melanoma cells (A375 n=4, SKMEL28 n=3 biological replicates of adipocyte secretome) treated with control (control, white), young (YA, blue), and old (OA, red) adipocyte secretome. Boxplot whiskers represent minimum and maximum values, box represents 25th and 75th percentiles, line represent median, two-way ANOVA.

(D) Box plot of basal respiration of melanoma cells (A375, SKMEL28) after exogenous mitochondrial transfer, central line is median, box is 25th to 75th percentile, whisker boundaries are minimum and maximum observed values, two-way ANOVA (n=5 biological replicates of adipocyte secretome).

(E) Mitochondrial ROS production in A375 cells exposed to control (control, black), young (YA, blue), and old (OA, red) adipocyte secretomes over 24 hours. Data represents mean and standard error, Kruskal Wallis Test (n=3 biological replicates of adipocyte secretome).

(F) Mitochondrial ROS production in SKMEL28 cells exposed to control, YA, OA adipocyte secretomes over 24 hours. Data represents mean and standard error, Kruskal Wallis test (Control-SKMEL28 n=3 (black), YA-SKMEL28 n=6 (blue), OA-SKMEL28 n=3 (red). All biological replicates of adipocyte secretome.

(G) Mitochondrial ROS production in MeWo cells exposed to control, YA, OA adipocyte secretomes over 24 hours. Data are mean and standard error, Kruskal Wallis test (Control-MeWo n=5 (black), YA-MeWo n=6 (blue), OA-MeWo n=6 (red)). All biological replicates of adipocyte secretome.

(H) Glycolysis (extracellular acidification rate, ECAR) of SKMEL28, A375 melanoma cells treated with young (YA, blue) and old adipocyte (OA, red) secretomes. Dot plots represent individual experimental measurements, lines are mean and standard error (A375 n=6; SKMEL28 n=5), two-sided Mann Whitney U. All biological replicates of adipocyte secretome.

(I) Basal respiration quantified through oxygen consumption rate (OCR) in melanoma cell lines, A375 (n=5), SKMEL28 (n=5), and MeWo (n=5) treated with either Young (YA, blue) or Young Lipid stripped (YA-Lipid Strip, white circles, blue outline) secretomes. Data are mean and standard error, two-way ANOVA. All biological replicates of adipocyte secretome.

(J,K) Relative confluence of (J) A375 cells (n=4) and relative confluence of (K) MeWo cells (n=3) treated with young (YA, blue) and YA lipid stripped secretome (YA-Lipid strip, blue boxed). Data are mean confluence and standard error, Kruskal Wallis test. All biological replicates of adipocyte secretome.

(L,M) Intracellular ROS levels of (L) A375 cells (n=3) and intracellular ROS levels of (M) SKMEL28 cells (n=3) treated with (Control, black), young (YA, blue), YA lipid stripped secretome exposure (YA-lipid strip, blue, squares). Data represent mean and standard error, Kruskal Wallis test. All biological replicates of adipocyte secretome.

(N) Intracellular ROS levels of MeWo cells (Control, black), young (YA, blue), or old adipocyte (OA, red) secretome exposure, YA lipid stripped secretome exposure (YA-lipid strip, blue, squares). Data are mean and standard error, Kruskal Wallis test (MeWo control n=4, YA-MeWo n=3, YA-MeWo-Lipid-Strip n=7). All biological replicates of adipocyte secretome.

(O) Box plots of the ratio of reduced (GSH) and oxidised (GSSG) glutathione in melanoma cell lines (A375, SKMEL28, MeWo) after treatment with young (YA, blue), or old adipocyte (OA, red) secretomes, or serum free media control (white) before and after stripping lipids (dotted and blue background), Boxplot whiskers represent minimum and maximum values, box represents 25th and 75th percentiles, line represent median, 2-way-ANOVA (Control-A375 n=5, OA-A375 n=6, YA-A375 n=7, A375-Lipid Strip n=5, OA-A375-Lipid Strip n=6, YA-A375-Lipid Strip n=4, Control-SKMEL28 n=6, OA-SKMEL28 n=6, YA-SKMEL28 n=5, SKMEL28-Lipid Strip n=8, OA-SKMEL28-Lipid Strip n=6, YA-SKMEL28-Lipid Strip n=6, Control-MeWo n=4, OA-MeWo n=4, YA-MeWo n=2, MeWo-Lipid Strip n=4, OA-MeWo-Lipid Strip n=4, YA-MeWo-Lipid Strip n=2). All biological replicates of adipocyte secretome.

(P) Relative confluence of A375 cells treated with young secretome (YA, blue) and YA secretome containing 10μM Etomoxir (YA Etomoxir, white circles). Data are mean relative confluence and standard error, two-sided Mann Whitney U (Control-A375 n=3, YA-A375 n=4, YA Etomoxir n=4). All biological replicates of adipocyte secretome.

(Q) Mitochondrial ROS in A375 cells treated with young secretome (YA, blue) and YA secretome containing 10μM Etomoxir (YA Etomoxir, white circles). Data are mean and standard error, Kruskal Wallis test (n=3). All biological replicates of adipocyte secretome.

(R) Western blot image of A375 and SKMEL28 cells transfected with shCPT1A and tubulin control.

(S) Proliferation of A375 cells and A375-shCPT1a knocked down cells treated with young (YA, blue), old adipocyte (OA, red) secretomes for 48 hours. Data are mean confluence and standard error, Kruskal Wallis test (n=2, biological replicates of adipocyte secretome).
(T) Proliferation of SKMEL28 control SKMEL28-shCPT1a knocked down cells treated with young (YA, blue), old adipocyte (OA, red) secretomes for 48 hours. Data are mean confluence and standard error, Kruskal Wallis test (n=2 biological replicates of adipocyte secretome).
(U) Basal respiration of A375, A375-shCPT1a, SKMEL28 and SKMEL28-shCPT1a treated with young (YA, blue) and old (OA, red) adipocyte secretomes. Data are mean with standard error, two-sided Mann Whitney U (n=2 biological replicates of adipocyte secretome).
(V) ATP production of A375, A375-shCPT1a, SKMEL28 and SKMEL28-shCPT1a treated with young (YA, blue) and old (OA, red) secretomes. Data are mean with standard error, two-sided Mann Whitney U (n=2 biological replicates of adipocyte secretome).

FIGURE S3

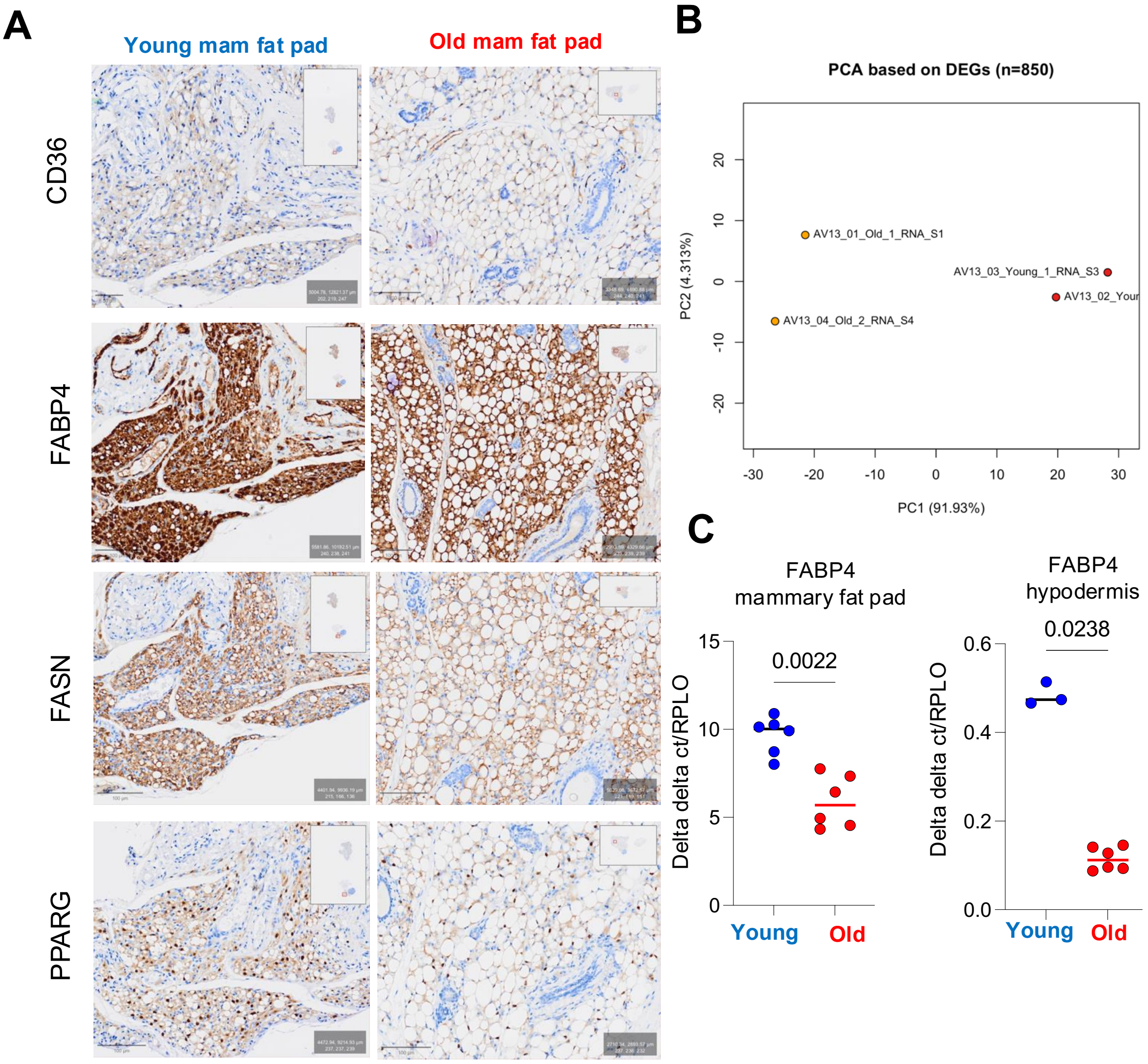

Figure S3: Murine mammary fat pad replicates hallmarks of murine aged hypodermis (Related to Figure 3)

(A) Images represent expression of adipogenic proteins (CD36, FABP4, FASN, PPARγ) in murine mammary fat pad.

(B) PCA plot of murine adipocytes showing the differences in transcriptomes of young and old murine mammary fat pad adipocytes.

(C) Graph represents the expression level of FABP4 in murine mammary fat pad and hypodermis with respect to RPLO housekeeping gene**.** Line represents mean and dots individual data points, two-sided Mann Whitney U (n=2 biological replicates, 3 technical replicates per sample).

FIGURE S4

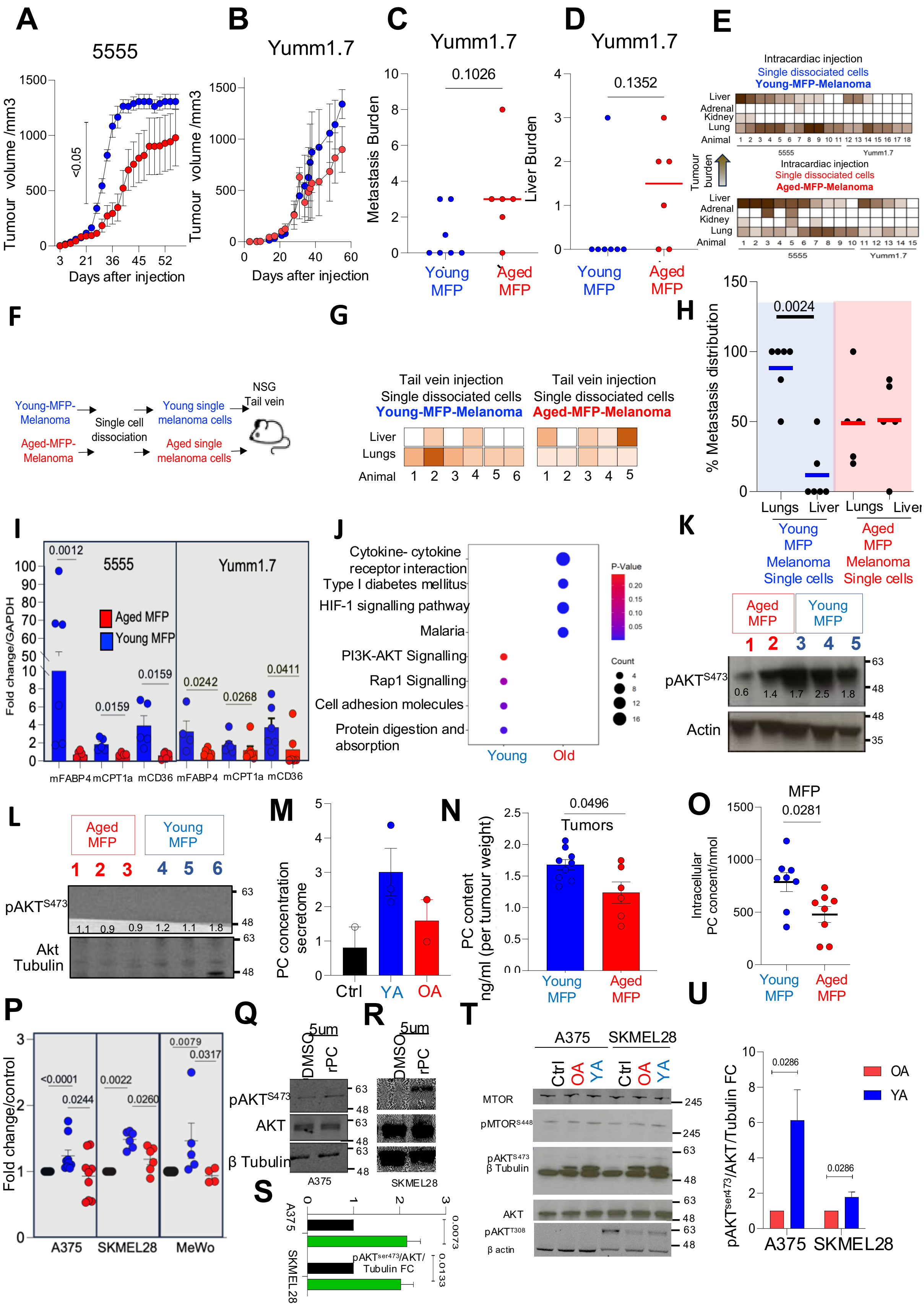

A
5555
Tumour volume /mm3
Days after injection
B
Yumm1.7
C
Yumm1.7
0.1026
Metastasis Burden
Young MFP
Aged MFP
D
Yumm1.7
0.1352
Liver Burden
E
Intracardiac injection
Single dissociated cells
Young-MFP-Melanoma
Aged-MFP-Melanoma
Liver
Adrenal
Kidney
Lung
Animal
Tumour burden
F
Young-MFP-Melanoma
Aged-MFP-Melanoma
Single cell dissociation
Young single melanoma cells
Aged single melanoma cells
NSG
Tail vein
G
Tail vein injection
Single dissociated cells
H
0.0024
% Metastasis distribution
Lungs
Liver
Young MFP Melanoma Single cells
Aged MFP Melanoma Single cells
I
0.0012
5555
Yumm1.7
Aged MFP
Young MFP
Fold change/GAPDH
mFABP4 mCPT1a mCD36 mFABP4 mCPT1a mCD36
J
Cytokine- cytokine receptor interaction
Type I diabetes mellitus
HIF-1 signalling pathway
Malaria
PI3K-AKT Signalling
Rap1 Signalling
Cell adhesion molecules
Protein digestion and absorption
Young
Old
P-Value
Count
K
pAKT S473
Actin
L
Akt
Tubulin
M
PC concentration secretome
Ctrl
YA
OA
N
0.0496
Tumors
PC content ng/ml (per tumour weight)
O
MFP
0.0281
Intracellular PC concent/nmol
P
Fold change/control
A375
SKMEL28
MeWo
Q
5um
DMSO
rPC
pAKT S473
AKT
β Tubulin
R
S
pAKTser473/AKT/Tubulin FC
T
MTOR
pMTOR S448
pAKT S473
β Tubulin
pAKT T308
β actin
U
pAKTser473/AKT/Tubulin FC
OA
YA

Figure S4: The primary tumor microenvironment dictates metastatic burden and tropism (Related to Figures 3, 4, 5)

(A) Individual growth curves of Young-MFP-5555 tumors, Aged-MFP-5555 tumors.

(B) Young-MFP-Yumm1.7 tumors, Aged-MFP-Yumm1.7 tumors. Data represent mean tumor growth volume with standard error, multiple t test (5555 n=8, Yumm1.7 n=4).

(C) Total tumor metastatic burden in C57BL/6 animals with Young-MFP-Yumm1.7 and Aged-MFP-Yumm1.7 melanomas. Data points represent mice and line represents mean, two-sided Mann Whitney U (Young-MFP-Yumm1.7 n=7; Aged-MFP-Yumm1.7 n=6).

(D) Liver metastases in C57BL/6 animals with Young-MFP-Yumm1.7 and Aged-MFP-Yumm1.7 tumors. Data points represent mice and line represents mean, two-sided Mann Whitney U (Young-MFP-Yumm1.7 n=7; Aged-MFP-Yumm1.7 n=6).

(E) Tile plot representing the metastasis burden in NSG mice injected with single dissociated cells from Young-MFP-5555, Aged-MFP-5555, Young-MFP-Yumm1.7, Aged-MFP-Yumm1.7 tumors.

(F) Experimental outline for tail vein injection experiments.

(G) Tile plot representing the metastasis burden in NSG mice injected with single dissociated melanoma cells from Young-MFP-Yumm1.7 and Aged-MFP-Yumm1.7 by tail vein.

(H) Metastasis distribution in the lungs and liver of mice injected with single melanoma cells from Young-MFP-Yumm1.7 (n=6) and Aged-MFP-Yumm1.7 (n=5) tumors. Data points represent mice and line represents mean, Kruskal Wallis test.

(I) Gene expression analysis of key genes associated with uptake (*CD36*), transport (*FABP4*), and metabolism of lipids (*CPT1a*) in Young-MFP-5555, Young-MFP-Yumm1.7, Aged-MFP-5555 and Aged-MFP-Yumm1.7 tumors. Graph represents the fold change compared to housekeeping genes GAPDH. Data are mean with standard error, two-sided Mann Whitney U (Young-MFP 5555 n=7, Aged-MFP 5555 n=6, Young-MFP Yumm1.7 n=7, Aged-MFP Yumm1.7 n=7)).

(J) Dot plot analysis representing the different pathways expressed in YA-MeWo, OA-MeWo (Young adipocytes n=1, Old adipocytes n=2 biological replicates).

(K) Western blot of $pAKT^{S473}$ and actin in mouse melanoma cells (5555) grown in C57BL/6 mammary fat pad of young and aged mice.

(L) Western blot of $pAKT^{S473}$, total Akt and tubulin in mouse melanoma cells (Yumm1.7) grown in C57BL/6 mammary fat pad of young and aged mice.

(M) Concentration of phosphatidylcholine (PC, µM) calculated with lipidomics in control, young and aged adipocyte secretome. Data represents mean with standard error (Control n=2 (black), YA n=3 (blue), OA n=2 (red)). Biological replicates.

(N) PC amount in nmol in the tumor grown in Young MFP and Aged MFP C57Bl/6 mice. Data represents mean normalized for the total MFP weight and standard error, two -sided Mann Whitney U (Young-MFP n=9 (blue), Aged-MFP n=6 (red)).

(O) PC amount in Young-MFP-5555 (n=8, blue) and Aged-MFP-5555 (n=8, red) tumors. Data represents the mean with standard error.

(P) PC fold change in melanoma cells following control media (black), young (YA, blue), old (OA, red) secretome exposure. Data represents the mean with standard error (Control-A375 n=9, YA-A375 n=9, OA-A375 n=9, Control-SKMEL28 n=6, YA-SKMEL28 n=6, OA-SKMEL28 n=6, Control-MeWo n=5, YA-MeWo n=5, OA-MeWo n=4). All biological replicates of adipocyte secretome.

(Q,R) Western blot of $pAKT^{S473}$, total AKT and tubulin in (Q) A375 and (R) SKMEL28 cells after serum free media control (DMSO) or 5µM PC exposure (rPC).

(S) Quantification of western blot analysis of $pAKT^{ser473}$, total AKT and tubulin in A375 and SKMEL28 melanoma cells after PC. Data represents mean with standard error, two-way ANOVA (n=2).

(T) Western blot of pmTORC2, pAKTser473, total AKT, $pAKT^{T308}$, mTOR, β tubulin and βactin in melanoma cell lines (A375, SKMEL28) treated with control, young adipocyte secretome (YA), and old adipocyte secretome (OA).

(U) Quantification of western blot analysis of $pAKT^{ser473}$, total AKT and tubulin. Data represents fold change mean with standard error, two-sided Mann Whitney U (n=4).

FIGURE S5

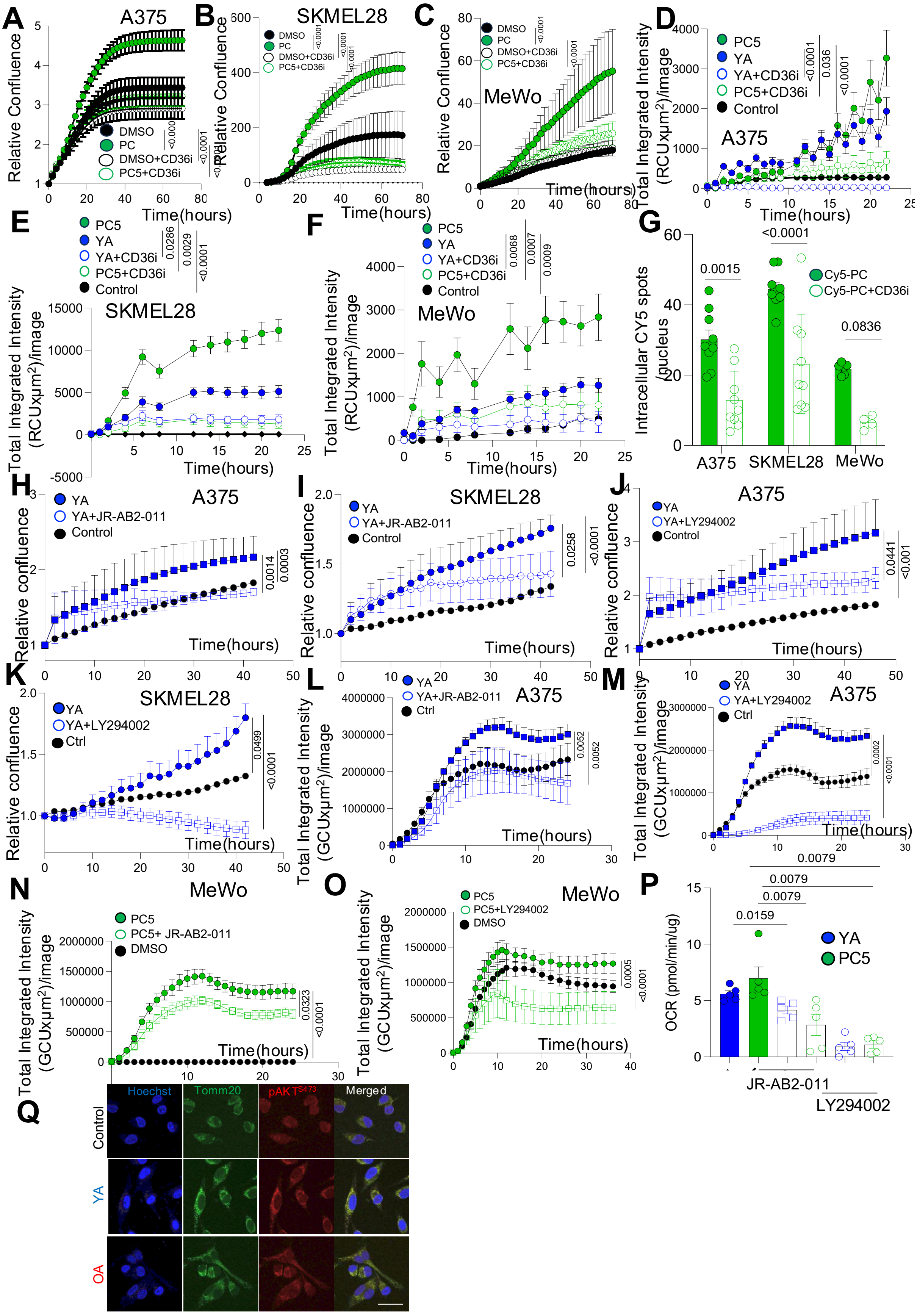
A
A375
Relative Confluence
DMSO
PC
DMSO+CD36i
PC5+CD36i
Time(hours)
B
SKMEL28
Relative Confluence
DMSO
PC
DMSO+CD36i
PC5+CD36i
Time(hours)
C
MeWo
Relative Confluence
DMSO
PC
DMSO+CD36i
PC5+CD36i
Time(hours)
D
A375
Total Integrated Intensity (RCUxµm2)/image
PC5
YA
YA+CD36i
PC5+CD36i
Control
<0.0001
0.036
<0.0001
Time(hours)
E
SKMEL28
Total Integrated Intensity (RCUxµm2)/image
PC5
YA
YA+CD36i
PC5+CD36i
Control
0.0286
0.0029
<0.0001
Time(hours)
F
MeWo
Total Integrated Intensity (RCUxµm2)/image
PC5
YA
YA+CD36i
PC5+CD36i
Control
0.0068
0.0007
0.0009
Time(hours)
G
Intracellular CY5 spots /nucleus
Cy5-PC
Cy5-PC+CD36i
0.0015
<0.0001
0.0836
A375
SKMEL28
MeWo
H
A375
Relative confluence
YA
YA+JR-AB2-011
Control
0.0014
0.0003
Time(hours)
I
SKMEL28
Relative confluence
YA
YA+JR-AB2-011
Control
0.0258
<0.0001
Time(hours)
J
A375
Relative confluence
YA
YA+LY294002
Control
0.0441
<0.001
Time(hours)
K
SKMEL28
Relative confluence
YA
YA+LY294002
Ctrl
0.0499
<0.0001
Time(hours)
L
A375
Total Integrated Intensity (GCUxµm2)/image
YA
YA+JR-AB2-011
Ctrl
0.0052
0.0052
Time(hours)
M
A375
Total Integrated Intensity (GCUxµm2)/image
YA
YA+LY294002
Ctrl
0.0002
<0.0001
Time(hours)
N
MeWo
Total Integrated Intensity (GCUxµm2)/image
PC5
PC5+ JR-AB2-011
DMSO
0.0323
<0.0001
Time(hours)
O
MeWo
Total Integrated Intensity (GCUxµm2)/image
PC5
PC5+LY294002
DMSO
0.0005
<0.0001
Time(hours)
P
OCR (pmol/min/ug)
YA
PC5
0.0079
0.0079
0.0079
0.0159
JR-AB2-011
LY294002
Q
Hoechst
Tomm20
pAKT S473
Merged
Control
YA
OA

Figure S5: Young lipids and phosphatidylcholine are taken up by melanoma cells and increase melanoma proliferation, ROS and respiration (Related to Figure 5)

(A,B,C). Relative proliferation of (A) A375 cells, (B) SKMEL28 cells and (C) MeWo cells treated with DMSO (black) 5μM Cy5-PC (green), DMSO and 20 μM CD36 inhibitor Salvianolic acid B (white with black outline) and Cy5-PC and 20 μM CD36 inhibitor (white circle with green outline). Data represents mean and standard error, n=3 replicates, two-sided Mann Whitney U.

(D,E,F) Mitochondrial ROS produced by (D) A375 cells, (E) SKMEL28 cells and (F) MeWo cells after control media (black, A375 n=3, SKMEL28 n=3, MeWo n=4); Young adipocyte exposure (blue, YA-A375 n=4, YA-SKMEL28 n=3, YA-MeWo n=5); Young adipocyte exposure and CD36 inhibitor (white, blue circle, YA+CD36i-A375 n=3, YA+CD36i-SKMEL28 n=3, YA+CD36i-MeWo n=3), PC5 exposure (green, PC-A375 n=5, PC-SKMEL28 n=6, PC-MeWo n=6), and PC5 exposure and CD36 inhibitor (white with green circle, PC5+CD36i-A375 n=6, PC5+CD36i-SKMEL28 n=6, PC5+CD36i-MeWo n=6). Data represents mean value with standard error, two-sided Mann Whitney U.

(G) Quantification of intracellular Cy5-PC in melanoma cell lines (A375, SKMEL28, MeWo) after treatment with 1μM Cy5-PC (green), and Cy5-PC and 20 μM CD36 inhibitor; data represents mean and standard error, two-sided Mann Whitney U (A375 n=9; SKMEL28 n=9; Cy5-PC-MeWo n=6, CY5-PC5+CD36i-MeWo, n=4).

(H, I). Relative proliferation of (H) A375 cells and relative proliferation of (I) SKMEL28 cells treated with young adipocyte secretome (YA, blue) or YA with 500nM mTORC2 inhibitor JR-AB2-011 (YA+JR-AB2-011, white square with blue outline) or control (black) for 48 hours (n=3 each condition). Data represents mean confluence and standard error, two-sided Mann Whitney U.

(J, K) Relative proliferation of (J) A375 cells and (K) SKMEL28 cells treated with young adipocyte secretome (YA, blue) or YA and 5μg/ml pAKT inhibitor LY294002 (YA+LY294002, white square with blue outline), and control (black) for 48 hours. Data represents mean confluence and standard error, two-sided Mann Whitney (A375-Control n=4, YA-A375 n=4, YA-A375+LY294002, n=4, SKMEL28-Control n=6, YA-SKMEL28 n=8, YA-SKMEL28+LY294002, n=8).

(L) Quantification of mitochondrial ROS in A375 cells after exposure to young adipocyte secretome (YA, blue), YA and 100nM mTORC2 inhibitor JR-AB2-011 (YA+JR-AB2-011, blue outline, white square), and control (black). Data represents mean and standard error of n=3 replicates all conditions, two-sided Mann Whitney U.

(M) Quantification of mitochondrial ROS in A375 cells after exposure to young adipocyte secretome (YA, blue), YA and 5μg/ml pAKT inhibitor LY294002 (YA+LY294002, blue line, white square). Data represents mean and standard error of n=3 replicates, two-sided Mann Whitney U.

(N) Quantification of mitochondrial ROS in MeWo after treatment with 5μM PC (PC, green), 5μM PC and 100nM mTORC2 inhibitor JR-AB2-011 (PC+JR-AB2-011, green outline, white square) and control (black). Data represents mean and standard error of n=3 replicates each condition, two-sided Mann Whitney U.

(O) Quantification of mitochondrial ROS in MeWo after treatment with 5μM PC (PC, green), 5μM PC and 5μg/ml pAKT inhibitor LY294002 (PC+LY294002) and control (black). Data represents mean and standard error, two-sided Mann Whitney U (MeWo-Control n=3, PC-MeWo +LY294002 n=4, PC-MeWo n=4).

(P) Mitochondrial basal respiration quantified through oxygen consumption rate (OCR) in SKMEL28 after treatment with young adipocyte secretome (YA, blue), 5μM PC (PC, green), YA and 100nM mTORC2 inhibitor JR-AB2-011 (YA+ JR-AB2-011. Blue outline, white), YA and 5μg/ml pAKT inhibitor LY294002 (YA+ LY294002. Green outline, white). Data represents mean and standard error of n=5 replicates each condition, two-sided Mann Whitney U.

(Q) Representative image of colocalization of $pAKT^{S473}$ (red), and mitochondria (Tomm20, green) in A375 cells treated with serum free media (control), young (YA) and old adipocyte (OA) secretomes. Scale bar 10 μm.

FIGURE S6

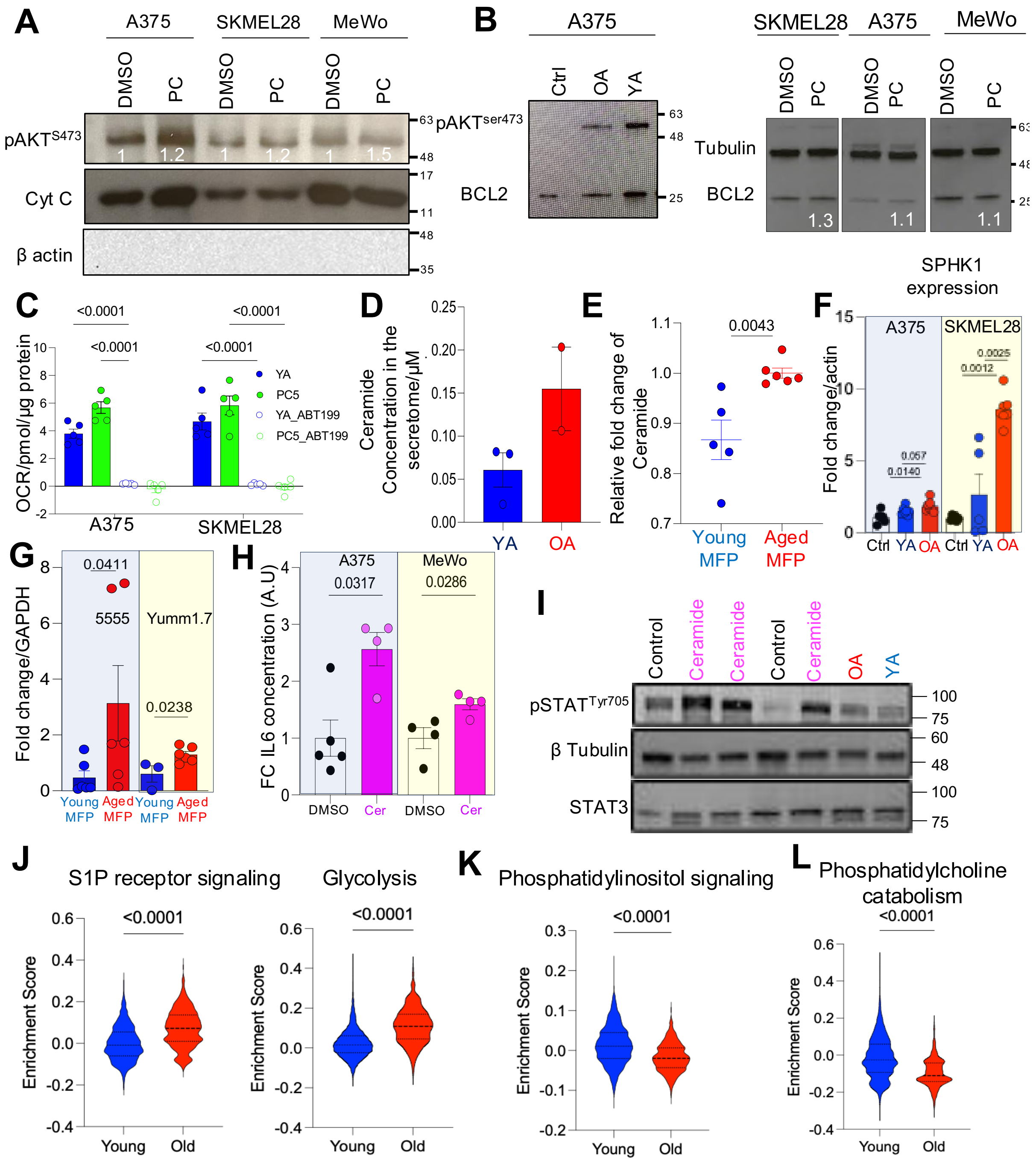
A
A375
SKMEL28
MeWo
DMSO
PC
pAKTS473
Cyt C
β actin
B
Ctrl
OA
YA
pAKTser473
BCL2
Tubulin
SPHK1
expression
C
OCR/pmol/µg protein
YA
PC5
YA_ABT199
PC5_ABT199
<0.0001
D
Ceramide Concentration in the secretome/µM
E
Relative fold change of Ceramide
0.0043
Young MFP
Aged MFP
F
Fold change/actin
G
Fold change/GAPDH
0.0411
5555
Yumm1.7
0.0238
H
FC IL6 concentration (A.U)
0.0317
0.0286
Cer
I
Control
Ceramide
pSTATTyr705
β Tubulin
STAT3
J
S1P receptor signaling
Glycolysis
K
Phosphatidylinositol signaling
L
Phosphatidylcholine catabolism
Enrichment Score
Young
Old

Figure S6: Phosphatidylcholine activates PI3K, BCL-2 and mitochondrial respiration in melanoma cells. Ceramides activate S1P-STAT3-IL-6 in melanoma cells (Related to Figure 5)

(A) Western blot of mitochondrial $pAKT^{S473}$ and cytochrome C loading control in melanoma cell lines (A375, SKMEL28, MeWo) after treatment with PC (5µM) or DMSO. Lack of cytoplasmic protein actin in the mitochondrial fraction confirms purity.

(B) Western blot images of $pAKT^{S473}$ and BCL2 in the mitochondrial fraction of A375 cells treated with serum free media (Ctrl), old adipocyte secretome (OA), young adipocyte secretome (YA) and 5µM PC (PC). Western blot image of BCL2 and beta tubulin in total lysate of SKMEL28, A375, and MeWo cells treated with DMSO and PC. Quantification compares to beta tubulin.

(C) Mitochondrial basal respiration quantified through oxygen consumption rate (OCR) in melanoma cell lines (A375, SKMEL28) after treatment with young adipocyte secretome (YA, blue), 5µM PC (PC, green), 1µM BCL2 inhibitor (ABT199), data represents mean and standard error, n=5 replicates, two-way ANOVA.

(D) Quantification of ceramide in young adipocyte secretomes (YA, blue, n=3), and old adipocyte secretomes (OA, red, n=2). Data represents mean and standard error.

(E) Fold change in intracellular ceramide content in Young-MFP and Aged-MFP C57BL/6 mice. Data represents the mean with standard error, two-sided Mann Whitney U (Young-MFP n=5, Aged-MFP n=6).

(F) Gene expression level of sphingosine kinase 1 (SPHK1) in melanoma cell (A375, SKMEL28, MeWo) exposed to either control (black), young (YA, blue), and old (OA, red) secretome. Data represents the mean fold change with standard error, two-sided Mann Whitney U (A375-Control n=6, YA-A375 n=7, OA-A375 n=7; SKMEL28-Control n=6, YA-SKMEL28 n=5, OA-SKMEL28 n=7; MeWo-Control n=6, YA-MeWo n=7, OA-MeWo n=7).

(G) Gene fold change in expression of sphingosine kinase 1 (*SPHK1*) in tumors grown in Young-MFP-5555 (n=6), Young-MFP-Yumm1.7 (n=3), Aged-MFP-5555 (n=6) and Aged-MFP-Yumm1.7 (n=5) tumors (young blue, aged red). Data represents the mean fold change to GAPDH with standard error, two-sided Mann Whitney U.

(H) IL-6 in the secretome of melanoma cells (A375, MeWo) after exposure to 100nM of synthetic ceramide or DMSO. Data represents fold change. Data represents mean with standard error, two-sided Mann Whitney U (A375-DMSO n=5, Ceramide-A375 n=4; MeWo-DMSO n=4, Ceramide-MeWo n=4).

(I) Western blot image of expression of $pSTAT3^{Tyr705}$, total STAT and tubulin in melanoma cells (A375 and SKMEL28) exposed to ceramide 100nM, YA, OA secretome.

(J) Graphs represent the enrichment score in Sphingosine 1 Phosphate Signalling (S1P) and glycolysis in melanoma subcutaneous metastases from young (blue n=2) and old (red n=2) human patients[43]. Violin plots show 25th to 75th percentiles and median (n=2 biological replicates), Mann Whitney U.

(K) Graphs represent the enrichment score in Phosphatidylcholine Catabolism (PC catabolism), Phosphatidylinositol Signalling in melanoma subcutaneous metastases from young (blue n=2) and aged (red n=2) human patients, Violin plots show 25th to 75th percentiles and median, Mann Whitney U.

FIGURE S7

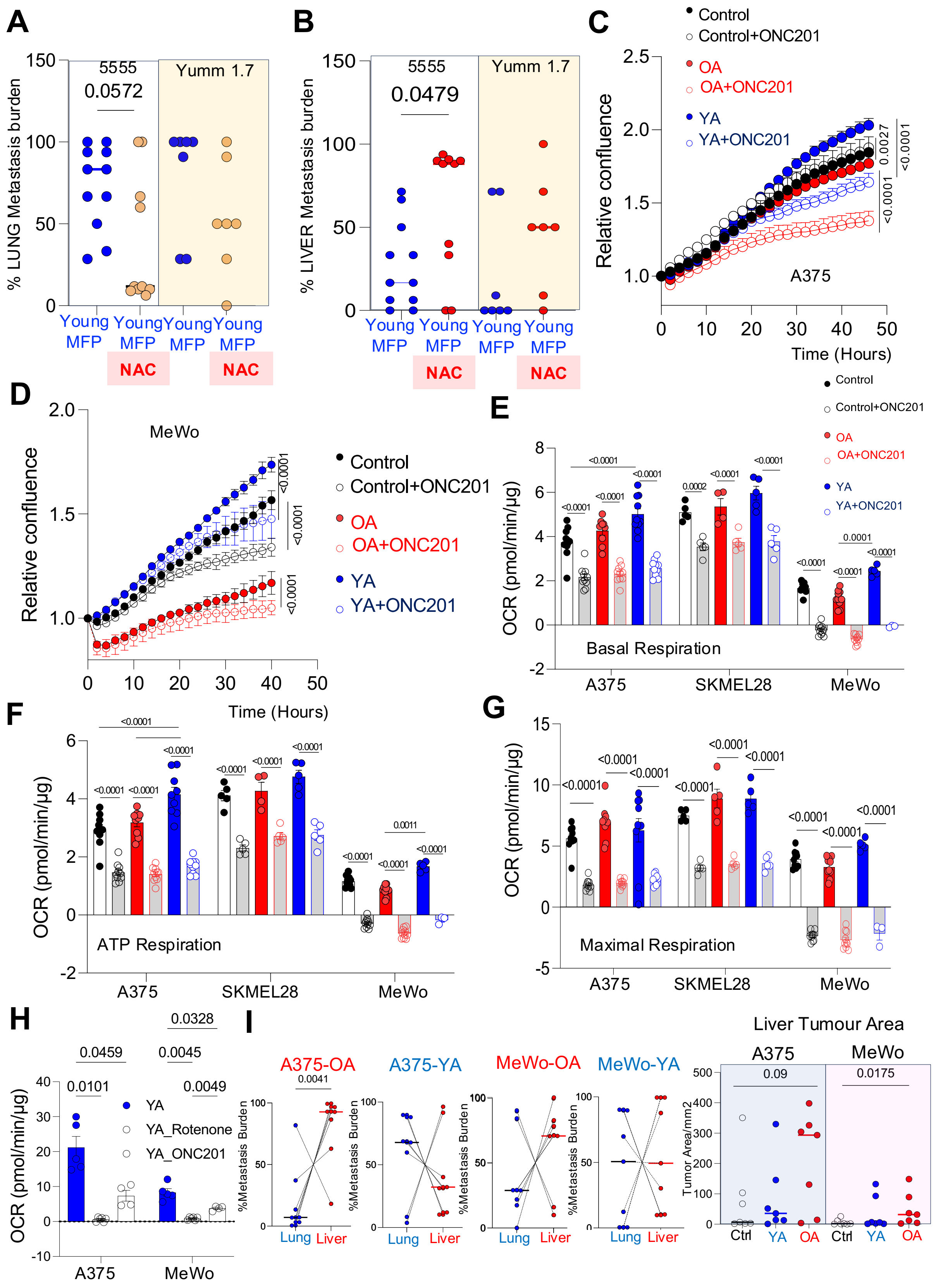
A
B
C
D
E
F
G
H
I
% LUNG Metastasis burden
% LIVER Metastasis burden
5555
Yumm 1.7
0.0572
0.0479
Young MFP
NAC
Relative confluence
Time (Hours)
Control
Control+ONC201
OA
OA+ONC201
YA
YA+ONC201
A375
MeWo
OCR (pmol/min/μg)
Basal Respiration
ATP Respiration
Maximal Respiration
SKMEL28
YA_Rotenone
YA_ONC201
A375-OA
A375-YA
MeWo-OA
MeWo-YA
%Metastasis Burden
Lung
Liver
Liver Tumour Area
Tumor Area/mm2
Ctrl
0.0041
0.09
0.0175
0.0459
0.0101
0.0045
0.0049
0.0328

Figure S7: OXPHOS levels dictate melanoma metastasis and tropism (Related to Figure 6)
(A) Lung metastasis distribution in animals after intracardiac injection of single dissociated cells from Young-MFP-melanomas (Young-MFP-5555 (n=11), Young-MFP-Yumm1.7 (n=7)) and Young-MFP-Melanomas supplemented with N-acetylcysteine (NAC) (Young-MFP-NAC-5555 (n=10), Young-MFP-NAC-Yumm1.7 (n=7). Dots are lung burden in individual animals, lines are the median, two-sided Mann Whitney U.
(B) Liver metastasis distribution in animals after intracardiac injection of single dissociated cells from Young-MFP-melanomas (Young-MFP-5555 (n=11), Young-MFP-Yumm1.7 (n=7)) and Young-MFP-melanomas supplemented with N-acetylcysteine (NAC) (Young-MFP-NAC-5555 (n=10), Young-MFP-NAC-Yumm1.7 (n=7). Dots are liver burden in individual animals, lines are the median, two-sided Mann Whitney U.
(C,D) Relative proliferation of (D) A375 cells (n=4) and relative proliferation of (E) MeWo cells (n=3) after media exposure (black), Young adipocyte (YA, blue), Old adipocyte (OA, red) secretome exposure and ONC201 3µM (control+ONC201, white circle, black outline; YA+ONC201, white circle, blue outline; OA+ONC201, white circle, red outline). Data represents mean value with standard error, two-way ANOVA.
(E) Basal respiration of melanoma cells exposed to media (black), Young adipocyte (YA, blue), Old adipocyte (OA, red) secretome and ONC201 3µM (control+ONC201, white circle, black outline; YA+ONC201, white circle, blue outline; OA+ONC201, white circle, red outline). Data represents mean value with standard error, two-way ANOVA, (A375-Control n=10, A375-Control+ONC201 n=10, OA-A375 n=9, OA-A375+ONC201 n=10, YA-A375 n=10, YA-A375+ONC201 n=10, SKMEL28-Control n=5, SKMEL28-Control+ONC201 n=5; OA-SKMEL28 n=4, OA-SKMEL28+ONC201 n=5, YA-SKMEL28 n=5, YA-SKMEL28+ONC201 n=5; MeWo-Control n=10, MeWo-Control+ONC201 n=10, OA-MeWo n=10, OA-MeWo+ONC201 n=10, YA-MeWo n=5, YA-MeWo n=3).
(F) ATP production of melanoma cells exposed to media (black), Young adipocyte (YA, blue), Old adipocyte (OA, red) secretome and ONC201 3µM (control+ONC201, white circle, black outline; YA+ONC201, white circle, blue outline; OA+ONC201, white circle, red outline). Data represents mean value with standard error, two-way ANOVA, (A375-Control n=10, A375-Control+ONC201 n=10, OA-A375 n=9, OA-A375+ONC201 n=10, YA-A375 n=9, YA-A375+ONC201 n=10, SKMEL28-Control n=5, SKMEL28-Control+ONC201 n=5; OA-SKMEL28 n=4, OA-SKMEL28+ONC201 n=5, YA-SKMEL28 n=5, YA-SKMEL28+ONC201 n=5; MeWo-Control n=10, MeWo-Control+ONC201 n=10, OA-MeWo n=10, OA-MeWo+ONC201 n=10, YA-MeWo n=5, YA-MeWo n=3).
(G) Maximal respiration of melanoma cells exposed to media (black), Young adipocyte (YA, blue), Old adipocyte (OA, red) secretome and ONC201 3µM (control+ONC201, white circle, black outline; YA+ONC201, white circle, blue outline; OA+ONC201, white circle, red outline). Data represents mean value with standard error, two-way ANOVA, (A375-Control n=10, A375-Control+ONC201 n=10, OA-A375 n=10, OA-A375+ONC201 n=10, YA-A375 n=10, YA-A375+ONC201 n=10, SKMEL28-Control n=5, SKMEL28-Control+ONC201 n=5; OA-SKMEL28 n=5, OA-SKMEL28+ONC201 n=5, YA-SKMEL28 n=5, YA-SKMEL28+ONC201 n=5; MeWo-Control n=10, MeWo-Control+ONC201 n=10, OA-MeWo n=10, OA-MeWo+ONC201 n=10, YA-MeWo n=5, YA-MeWo n=3).
(H) Graph represents the mitochondrial basal respiration of A375 and MeWo cells when exposed to YA (blue circles, n=5), YA with rotenone 0.25µM (lighter blue circle n=5) and YA with ONC201 3µM (lightest blue circle n=4). Data represents mean with standard error mean, two-sided Mann Whitney U.
(I) Graphs represent HALO analysis of two studies to validate the metastatic scores used for the study.

Table S7: Oligonucleotides (qPCR primers sequence used in this study, related to STAR Methods)

Gene	Forward Primer	Reverse Primer
h*FABP4*	5'-CCGGGAAACTGTGGCGTGATG-3'	5'-AGGTGGAGGAGTGGGTGTCGCTGTT-3'
hADIPOQ	5'-CTGTTGCTGGGAGCTGTTCT-3'	5'-CCCTTAGGACCAATAAGACCTGG -3
h*FASN*	5'-CAACCTCTCCCAGGTATGCG-3'	5'-ACCCTTCAATCCCGTTGCAT-3'
h*PPARG*	5'-GCAAACCCCTATTCCATGCTG-3'	5'-ACCCTTCAATCCCGTTGCAT-3'
hSCD	5'-TTCCCGACGTGGCTTTTTCT-3'	5'-AGCCAGGTTTGTAGTACCTCC-3'
hSPHK1	5'-GCTGCGAAGTTGAGCGAAAA-3'	5'- GGCTGGACCCAGTCGG-3'
hCPT1a	5' TTTGGACCGGTTGCTGATGA-3'	5'- TTTCCAGCCCAGCACATGAA-3'
hCD36	5'-ACTGAGGACTGCAGTGTAGGA-3'	5'- ACAAGCTCTGGTTCTTATTCACA-3'
h*GAPDH*	5'-CCGGGAAACTGTGGCGTGATG-3'	5'-AGGTGGAGGAGTGGGTGTCGCTGTT-3'
hactin	5'-ACA GAG CCT CGC CTT TGC -3'	5'-CCA CCA TCA CGC CCT GG -3'
mCPT1a	5'- GACTCCGCTCGCTCATTCC -3'	5'ACCAGTGATGATGCCATTCTTG-3'
mCD36	5'-TGTGGAGCAACTGGTGGATG-3'	5'-CGTGGCCCGGTTCTAATTCA-3'
mSPHK1	5'-TTTGGAGGTTGCTGACGAGG-3'	5'-CGGGGCGGCCAGATTTTTAG-3'
mFABP4	5'- TGA TGC CTT TGT GGG AAC CT -3'	5'- CCC GCC ATC TAG GGT TAT GA -3'
mRPLO	5'- GGC GAC CTG GAA GTC CAA CT -3'	5'- CCA TCA GCA CCA CAG CCT TC -3'
